# Incremental Value of Smartphone Sensing for Monitoring Momentary Affect Intensity in Adults Using Transformer-Based Models: Observational Study

**DOI:** 10.2196/90970

**Published:** 2026-09-28

**Authors:** Yiqin Zhu, Yuyi Yang, Renee J Thompson

**Affiliations:** 1 Department of Psychological and Brain Sciences, Washington University in St. Louis, 1 Brookings Drive, CB 1125, St. Louis, MO, 63130, United States, +1-314-935-3502; 2 Division of Computational and Data Sciences, Washington University in St Louis, 1 Brookings DriveSt. Louis, MO, 63130, United States

**Keywords:** transformer-based models, affect, smartphone sensing, ubiquitous computing, affective computing, digital phenotyping

## Abstract

**Background:**

Ubiquitous smartphone access and statistical advances offer opportunities to continuously track affect intensity, which is central to various psychological processes and behaviors. Research demonstrated the potential of personalized predictions of momentary negative affect (NA) and positive affect (PA) using passive sensing. However, studies typically incorporated all available data sources without differentiating their added value, nor did they investigate whether refining location features with self-reported semantic location (eg, workplaces) improved personalized predictions.

**Objective:**

We evaluated three specific aims: (1) how different combinations of data sources improved performance compared to personalized baseline models, (2) whether model predictions differed across passive data aggregation timescales, and (3) whether incorporating self-reported semantic locations improved model predictions.

**Methods:**

Adults (final n=133) completed a 14-day ecological momentary assessment (EMA) protocol reporting emotional experiences 5 times daily alongside smartphone sensing. Testing data (n=532 EMAs) used the last 4 surveys for each individual, with the remaining used for training and validation (n=6805 [NA]/6800 [PA] EMAs). We evaluated whether combinations of personalization, passive sensing, and affect history improved baseline prediction, and how full-information temporal fusion transformers (TFTs) performed across 6 timescales (1, 3, 6, 12, 24, and 48 hours), with or without self-reported semantic location features.

**Results:**

The baseline model, using each individual’s mean affect in the training set, demonstrated moderate predictive performance for NA (mean absolute error [MAE]=0.66, 95% CI 0.60‐0.73; *R*²=40.2%) and PA (MAE=0.71, 95% CI 0.65‐0.78; *R*²=36.1%). Full-information TFTs improved NA prediction (MAE=0.62, 95% CI 0.56‐0.69; *R²*=45.2%; ΔMAE=−0.04, 95% CI −0.06 to −0.01; *P* values ≤.004; Cohen *d*=−0.27) but not PA prediction (MAE=0.70, 95% CI 0.64‐0.78; *R*²=32.5%; ΔMAE=−0.01, 95% CI -0.03 to 0.02; *P* values >.10; Cohen *d*=−0.04). No pairwise timescale comparison survived false discovery rate (FDR) correction (NA: *P*_FDR_=.05-.98; PA: *P*_FDR_=.08-.99). Adding self-reported locations did not improve NA prediction (ΔMAE=0.02, 95% CI −0.01 to 0.04; *P* values >.20; Cohen *d*=0.11) or PA prediction (ΔMAE=0.01, 95% CI −0.01 to 0.03; *P* values>.33; Cohen *d*=0.07). However, incorporating self-reported semantic locations changed the composition and relative ranking of important inputs, with these changes varying across NA and PA and between past and future inputs.

**Conclusions:**

Incorporating smartphone features provided a modest and significant improvement in momentary NA prediction, but not PA prediction. Model performance did not vary across passive data aggregation timescales. While adding self-report semantic locations did not improve prediction accuracy, it changed variable-importance patterns and may provide additional context for interpreting digital behavioral markers. Future personalized predictions should incorporate person-mean affect as an essential benchmark. These findings support passive smartphone sensing as a valuable supplement to, rather than a replacement for, active EMA.

## Introduction

### Background

Modern technologies already enable us to unobtrusively and accurately track sleep, physical activity, or health essentials such as heart rate and blood pressure, but what about affect intensities? Like the continuous tracking of human behaviors indicative of health status, continuous tracking of affect intensities holds broad research and health implications, given the central role of affect intensity in various outcomes (eg, attention [1], memory scope [2], and decision-making [3]) and its defining role in the diagnostic criteria of many mental disorders [4]. In evaluations for mood and anxiety disorders, for example, diagnostic impression relies on individuals’ retrospective reports of their affect intensity patterns compared to their own baseline patterns (eg, intense negative affect [NA] or blunted positive affect [PA]) over an extended period in the past (eg, the past several months or years). However, retrospective reports are affected by recall bias, especially in clinical samples [5,6]. Assessing the intensity of one’s subjective feelings from moment to moment reduces bias but requires constant attention that results in burden and missingness (eg, 30% missingness for data collection less than a month [7]). These limitations, bias in recalling experiences in the past, and the burden of subjective momentary assessment could be overcome by personalized models to passively and continuously compute affect intensities. Research suggested the potential of such personalized prediction using data from smartphone sensors [8-10]. However, existing studies combined all data sources in models without differentiating each source’s contribution, particularly between an individual’s own affective history and passively sensed behaviors. As a result, it remains unclear whether passive sensing meaningfully improves prediction beyond what could already be achieved from a person’s own affective history (eg, person-mean affect). The overarching aim of this study was to parse out variances of different data sources from personalized prediction models. In addition, we used linear mixed-effects models and post hoc interpretability measures (ie, attention weights) from machine learning (ML) to generate hypotheses about relations between affect and smartphone-tracked behaviors.

### Monitoring and Understanding Momentary Affect Intensity Through Smartphones

Cheap and wide access to smartphones (91% ownership in the United States [11]) provides unprecedented opportunities not only for passive monitoring of momentary affect intensity but also for understanding ubiquitous human-smartphone interactions. Five studies incorporated smartphone sensor data and modeled momentary affect intensities. Three of the studies dichotomized affective intensity as outcomes and achieved modest prediction with data from smartphones, smartwatches, and smartrings [8-10]. Research showed that traditional AI algorithms could make moderate predictions on whether an individual’s daily affect intensities [9] or momentary affect intensities [10] were above a certain threshold, and make just-surpassing-chance predictions of the presence of affect in unseen individuals [8]. However, dichotomizing affect intensity loses important granular information, as different levels of intensity of the same type of emotion lead to different behavioral outcomes. For example, strong feelings of anger might lead to physical violence, whereas weak feelings of anger might lead to verbal profanity or no overt behavior. Therefore, affect intensity is, by nature, continuous and, therefore, motivates approaches that preserve variation across a continuous scale.

Research that continuously modeled momentary affect included predictors that require individuals’ active engagement [12,13] and only focused on certain NA without modeling PA. One study predicted momentary depressed feelings with smartwatches and self-reported items [13]. Both momentary anxiety and depressed affect were reported at the same time, and anxiety was the most important predictor of momentary depressed affect in the models, above smartwatch predictors, for 11 out of 14 participants. The other study collected heart rate metrics (ie, participants pressed their finger against the rear camera for 30 seconds hourly), along with smartphone sensors, to predict momentary depressed mood [12]. Importantly, these models combined all sources and did not test against simple baselines of a person’s mean affect. Thus, it remains unclear how much smartphone sensing specifically adds to personalized prediction. Finally, it is equally important to test personalized PA prediction using smartphone sensing. Theoretical models [14] and clinical trials [15,16] have highlighted the critical role of raising PA in reducing depression, and PA is conceptually independent of NA [17] and influenced by different biopsychosocial predictors from NA [18-20].

Smartphones are both a data-collection tool people carry and an object to interact with regularly, but few studies have examined associations between momentary affect intensity and smartphone-tracked behaviors. People engage in social interactions (eg, making phone calls) and interact with phone screens for multiple purposes, including using social media. Revealing relations between everyday affective experiences and behaviors tracked by smartphones could inform intervention strategies to aid emotion regulation. For example, if total screen use time in the prior hour is an important predictor of feeling worse among other smartphone-tracked behaviors (eg, total distance traveled), individuals could practice limiting their screen usage. One challenge for elucidating affect–smartphone-based behavior associations (and probably for most psychological research) is the large number of smartphone-tracked behaviors and the dependencies between them. For example, when individuals are traveling (as collected through GPS), they may make fewer phone calls and have fewer screen interactions. On the other hand, when individuals remain at home for an extended time, their likelihood of unlocking their screens may be higher. Thus, smartphone-tracked behaviors can be predictors of each other and work together to shape affective experiences.

Temporal fusion transformer (TFT) is well-suited to address the challenge brought by multiple and interacting predictors. As a state-of-the-art algorithm for time-series analysis, TFT may predict momentary affect intensity more accurately than traditional AI models while addressing the challenge brought by multiple and interacting predictors, as shown in its performance for other time-series predictions (eg, electricity, traffic, retail, and volatility [21]). As a transformer-based model, TFT uses self-attention to capture dependencies across time, while its variable selection networks identify predictors that are most informative for the prediction task [21]. Additionally, TFT accommodates 3 types of inputs based on their temporal availability: static inputs (time-invariant inputs), past inputs (predictors before the time point of making predictions), and future inputs (inputs corresponding to prediction time points processed by the decoder [21]). TFT’s capacity to distinguish the 3 input types is important for predicting momentary affect intensity. Because affect intensity is often viewed as an individual difference characteristic [22], adding static inputs may enhance prediction, although there is high intraindividual variability for momentary affect intensity. Additionally, it remained unclear at which lag length momentary affect intensity can be predicted by different smartphone-tracked behaviors. While traditional psychological research testing temporal associations usually adopts one lagging length (eg, lagging one assessment schedule), TFT could integrate sensing features derived across multiple temporal windows, allowing information from both proximal and more distal timescales to contribute to prediction.

### Timescales and Semantic Locations

Passive sensing research showed that the timescales to aggregate passive sensing data may influence the interpretation of results on psychological well-being [23]. Research on predicting momentary affect with passive sensors has used different timescales [8-10,12,13], and there is a lack of empirical tests comparing the influence of timescales on model performance and predictor importance. For affect intensity, the same behavioral indicator from smartphone sensors with different timescales could have different clinical implications. Intuitively, staying stationary for most of the time in the past hour might be less influential on momentary affect intensities than staying stationary for most of the time in the past 48 hours. Thus, how TFTs performed differently across timescales of aggregating the smartphone sensor data warrants empirical investigation. The timescales might also change the attention that TFTs put on the mobility markers (as collected by GPS) and social interaction markers (as collected by phone calls). Since social interactions are acute events or stressors [24], proxies of social interactions tracked by smartphones may be important predictors for NA and PA intensities when they are aggregated in smaller timescales. By contrast, physical mobility patterns in shorter timescales may be influenced by random factors (eg, weather), whereas they may indicate behavioral routines when aggregated in larger timescales. Therefore, mobility patterns tracked by smartphones may be more important when aggregated on larger timescales.

Contextual features, such as the semantic meaning of a location to an individual (eg, where they work, or where they entertain, or where they spend time with important others), might improve both model performance and interpretations. Affective experiences are constructed by subjective evaluations of both internal and external cues [25,26]. A model of situation perception [27] proposes three situational cues: (1) persons and interactions (who?); (2) objects, events, and activities (what?); and (3) spatial location (where?). Physical locations recorded through GPS can provide information about “where” but not “what,” namely, the activities or the motivations for activities. Semantic meaning of a location may offer such information. For example, one may assume the major activities that individuals engage in are work in their self-reported workplaces, or that the major activities that they engage in are recreational in their self-reported leisure places. In prior studies, usually one semantic location feature (time spent at home) was included [11,28,29]. Collecting self-report locations from individuals at baseline may improve the coding accuracy of semantic locations and help create new location features, thereby potentially improving model performance. Additionally, semantic locations could inform intervention strategies, as they may elicit more awareness of what patients are engaging in than where they are. Overall, the role of semantic locations in predicting momentary affect warrants further investigation.

### Study Aims and Hypotheses

This study collected data from a sample of unselected adults who completed an ecological momentary assessment (EMA) protocol measuring self-report emotional experiences alongside continuous smartphone behavioral monitoring. For aim 1, we evaluated the incremental predictive value of contributions of personalization (participant identity and affective history) versus smartphone sensing data in momentary affect prediction, by comparing TFT and other ML models that vary in their usage of these data sources against a person-mean affect baseline. We hypothesized that the full TFT model incorporating all data sources would achieve prediction performance for unseen momentary NA and PA above the person-mean-affect baseline (hypothesis 1). For aim 2, we examined how feature extraction across 6 aggregation timescales (1, 3, 6, 12, 24, and 48 hours preceding the report) affected TFT’s performance. We explored the optimal aggregate windows (exploratory aim 1) and identified top behavioral predictors across timescales. We hypothesized that social interaction (eg, call logs) and screen activity would demonstrate greater feature importance at proximal timescales (hypothesis 2a), whereas mobility markers might be more important at longer timescales (hypothesis 2b). For aim 3, we examined the impact of integrating participant-annotated semantic location features (eg, work and home of significant other). We hypothesized that refining semantic location features with participant self-reports would improve model performance (hypothesis 3a) and shift variable importance rankings (hypothesis 3b). Because attention weights reflect predictive relevance rather than causal mechanisms, findings from hypotheses 2a, 2b, and 3b are framed as exploratory and hypothesis-generating.

## Methods

### Participants

A total of 179 adults from the greater area of St. Louis, Missouri, United States, participated in a study to understand daily emotional experiences [30,31]. Individuals were eligible if they had a smartphone and at least one active social media profile. They were ineligible if they had any contraindications for peripheral physiological assessment (eg, pregnant and with an implanted cardiac device). Participants with less than 40 valid EMAs (70 possible surveys) on NA or PA (n=46) were excluded from further analysis. As a result, 133 out of 179 (74.3% of original participants) were retained in final analysis. Although there are no recommendations or requirements on the number of time points within each individual, in general, more time points are better. The time points ranged from 90 to 252 in the original TFT paper [21]. Considering the sample size to be retained, we decided on the threshold of 40 EMAs such that the rate of sample size to be retained in this study (74.3%) was comparable to or higher than existing studies that predict momentary affect intensity (71.6% [8]; 35% [9]).

### Ethical Considerations

The study was approved by the Washington University Institutional Review Board (number 202209063) and was performed in line with the standards of the 1964 Declaration of Helsinki. Informed consent and assent were collected from all participants. To protect participant privacy, all data were deidentified before analysis and stored on secure, password-protected servers. Participants were compensated US $135 for completing the study. There was a US $15 bonus if participants completed 80% (56/70) surveys or more. Thus, participants can receive up to US $150 depending on their survey completion rates.

### Study Design and Procedure

Eligibility was determined via a phone screen. Participants were scheduled for an in-person laboratory session. During the laboratory session, participants completed a semistructured EMA tutorial, including a practice EMA survey, administered by a staff member or an undergraduate research assistant. The tutorial included helping participants download and install the SEMA^3^ app (developed and hosted by the Melbourne eResearch Group at the University of Melbourne [32]) for EMA surveys and the AWARE ([33]; University of Oulu) apps for smartphone sensing. EMA surveys occurred during a 15-hour window of the participants’ choice. Participants were surveyed 5 times a day for 14 days, starting the day after the laboratory session. At the laboratory session, participants also reported anticipated locations they frequently visited (eg, office and home) and their addresses in the next 14 days. The AWARE framework [33] is an open-source mobile app to unobtrusively record location coordinates, screen interactions (ie, when the screen status changed to on or off and locked or unlocked), call logs for incoming, outgoing, and missed calls, and Bluetooth connections. The AWARE started to collect deidentified data unobtrusively from the smartphone sensors after installation. By default, location coordinates are sampled once per 180 seconds, and Bluetooth is sampled once per 60 seconds. Calls and screen usage are event-based sensor streams. Bluetooth data were not included in analyses due to high missingness. A total of 156 out of 179 (87.2%) participants had no Bluetooth scans recorded in the hour preceding any EMA prompt, and a total of 103 out of 179 (57.5%) participants had no Bluetooth data at all.

The original sample completed a total of 8690 out of 12,530 (69.4%) EMA surveys. On average, individuals completed 49 out of 70 (70%; SD 15.1) EMA surveys. The completion rate was higher in the final analytic sample, which on average completed 56 out of 70 (80%) EMAs because of the selection criteria (see the “Participants” section). Participants could receive up to US $135 for completing the study and a bonus of US $15 if their EMA completion was more than 80%, resulting in a possible compensation of $150.

### Measures

#### EMA: Momentary Affect Intensity

In each survey, participants were asked to report on how they felt in the hour preceding the EMA survey. They indicated the extent to which they felt 4 PA and 4 NA using a 7-point Likert scale (1=not at all; 7=extremely). Items were chosen to represent a 2-dimensional affective circumplex (ie, valence and arousal). The PA items were “During the last hour, I felt CONTENT/CALM/HAPPY/ENTHUSIASTIC,” and the NA items were “During the last hour, I felt SAD/SLUGGISH/WORRIED/FRUSTRATED.” In the final analytic sample, both NA and PA showed acceptable within-person reliability (NA: *ω*_within_=0.64, PA: *ω*_within_=0.69) and great to excellent between-person reliability (NA: *ω*_between_=0.88, PA: *ω*_between_=0.90).

#### Measures Derived From Passive Sensors

##### Overview

Figure 1 illustrates the pipeline of data collection, cleaning, and analysis.

Table 1 presents a list of extracted features. We computed features from 3 smartphone sensors (location coordinates, screen interactions*,* and call logs) because of their potential to predict depression severity [28,29,34,35] or predict dichotomized momentary affect intensity [10].

**Figure 1.**
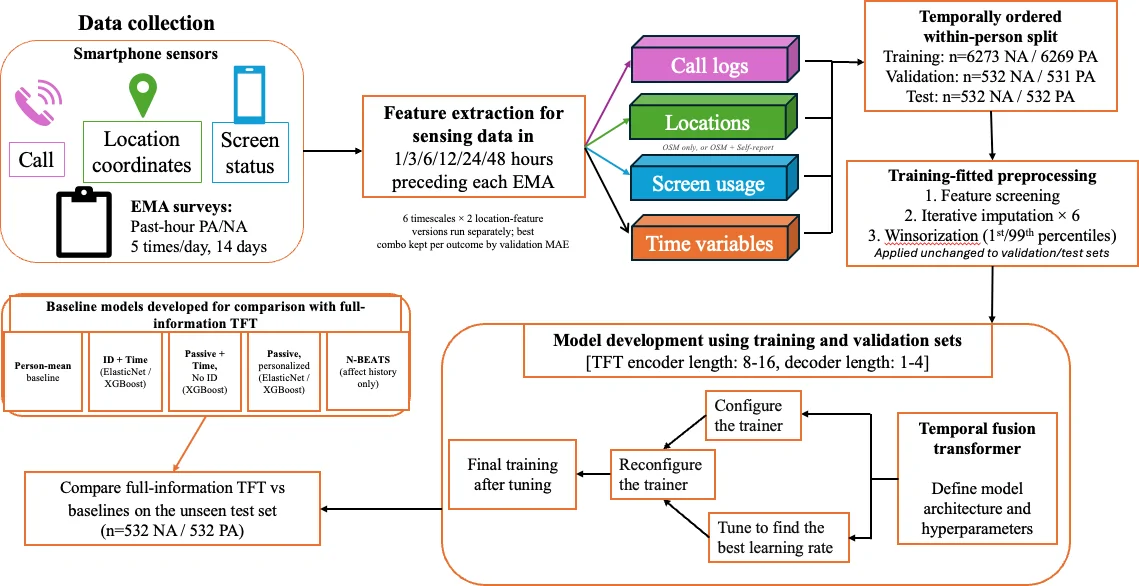
Data collection, cleaning, and analysis pipeline. EMA: ecological momentary assessment; ElasticNet: elastic net regression; MAE: mean absolute error; NA: negative affect; N-BEATS: neural basis expansion analysis for interpretable time series forecasting; PA: positive affect; TFT: temporal fusion transformer; XGBoost: extreme gradient boosting.

**Table 1.** Predictors derived from smartphone sensors and their sources^a^.

Variable type and sensor source	Variables
Spatial mobility markers	
GPS coordinates [28,34,35]	Location variance Log of location variance Total distance traveled Average speed Variance in speed Percentage of time being stationary (stationary: momentary speed <1 km/h) Radius of gyration
GPS coordinates clustered by DBSCAN^b^ [28,35]	Number of significant places Number of significant transitions Percentage of time spent in rarely visited locations (ie, outliers by DBSCAN) Location entropy Normalized location entropy
Clusters labeled by OSM^c^ or self-report locations	Percentage of time spent at home (OSM) Percentage of time spent at leisure places (OSM) Percentage of time spent at home (OSM + self-report) Percentage of time spent at leisure places (OSM + self-report) Percentage of time spent at workplaces (OSM + self-report) Percentage of time spent at home of significant others (OSM + self-report)
Proxies of smartphone social interactions	
Call logs [28]	Number of incoming calls Number of outgoing calls Number of missed calls Number of correspondents called Duration of incoming calls (average, maximum, minimum, and total duration) Duration of outgoing calls (average, maximum, minimum, and total duration)
Screen interactions	
Screen status [36]	Number of unlocked episodes Average, maximum, minimum, and total screen-on duration
Time variables	
Timestamp	Relative time in hours since the first self-report EMA^d^ survey Weekend or weekday (1=weekend; 0=weekday) Time of day (night: midnight to 6 AM; morning: 6 AM to noon; afternoon: noon to 6 PM; evening: 6 PM to midnight) Prompt (the number of the self-report EMA survey)

a Each predictor was computed for each timescale. For example, for the timescale of 3 hours, the location variance of the 3 hours preceding each survey was created.

b DBSCAN: Density-Based Spatial Clustering of Application with Noise.

c OSM: OpenStreetMap.

d EMA: ecological momentary assessment.

##### Location Feature Sets

Location features are derived from the coordinates of latitude and longitude and the timestamp of each coordinate. We first removed duplicated data points for each individual and then extracted the following location features consistent with research [28] that detected depression and predicted its onset from smartphone data: (1) location variance (sum of the variance in latitude and longitude coordinates), (2) log of location variance, (3) total distance traveled, (4) average speed, (5) variance in speed, (6) the percentage of time being stationary (stationary was defined as momentary speed being less than 1 km/h), and (7) radius of gyration. When calculating distances and speed, only adjacent data points whose time difference was greater than 1 second and less than twice the sampling rate (ie, 180 seconds) were retained.

Then each individual’s location coordinates were clustered using Density-Based Spatial Clustering of Applications with Noise (DBSCAN [37]), a clustering algorithm for large spatial databases with noise. Consistent with prior work [28], based on identified clusters, we extracted the (1) number of significant places, (2) the number of location transitions, (3) the percentage of time spent in insignificant or rarely visited locations (ie, outliers identified by DBSCAN), (4) location entropy (higher location entropy occurs when time is spent evenly across significant places), and (5) normalized location entropy across significant places (following previous definitions and operationalizations [35]).

Consistent with prior work [11,28,29], we assumed the place most visited by the participant late at night (between midnight and 6 AM) was their home location. Specifically, the location coordinates of the cluster most frequently used during all nights were assumed to be the participant’s home location center. Then, based on the coordinates of longitude and latitude for each individual’s core clusters, we labeled their type of location through the OpenStreetMap Nominatim API’s geocoding [38]. Locations labeled as “highway,” “aeroway,” “tourism,” “leisure,” and “shop” by OpenStreetMap were categorized as leisure and recreation. For each of the location labels (including the labels in the next paragraph), when participants’ GPS coordinates were within 100 meters of one location center cluster, their location at that timestamp was given the label of the location center cluster. For example, a participant was assumed to be at home if they were within 100 m of the home location. With these 2 labels, we extracted two location features: (1) the percentage of time spent at home and (2) the percentage of time spent at leisure and recreation.

We tested whether adding more semantic location features could improve model prediction and interpretation through their indication of possible activities. These sets of location features were extracted based on participants’ self-reported locations, which included categories of personal home, home of significant others (eg, parents, partner, and so on), workplace, and places for leisure and recreation. Based on their self-report addresses, the locations of the previously coded home and leisure were corrected, resulting in two extracted features: (1) the percentage of time spent at home (self-report) and (2) the percentage of time spent at leisure and recreation (self-report). Additionally, with 2 additional addresses (workplace and home of significant others), we extracted (3) the percentage of time spent at work (self-report) and (4) the percentage of time spent with significant others (self-report).

We created 6 sets of location features based on 6 temporal resolutions (ie, 1, 3, 6, 12, 24, and 48 hours) preceding each momentary affect (ie, NA and PA) report. Take the location variance feature as an example. We created 6 location variance features for a given momentary affect report—the location variance during 1, 3, 6, 12, 24, and 48 hours preceding the report.

##### Calls Feature Sets

Call features were calculated using the smartphone’s call logs. Consistent with prior work [28], we extracted the following six features: (1) the number of all incoming and outgoing calls, (2) the number of missed calls and the number of correspondents overall, and (3) the duration of all incoming and outgoing calls (in terms of their average, maximum, minimum, and total duration). As with the location features, we created 6 sets of call features based on 6 temporal resolutions (ie, 1, 3, 6, 12, 24, and 48 hours preceding each momentary affect report).

##### Screen Feature Sets

Screen features were calculated using the smartphone’s screen status sensor, which recorded screen status (ie, on or off) and its timestamp once the screen switched from one status (eg, on) to the other (eg, off). Consistent with prior work [28], we extracted the following five phone usage features: (1) the number of unlock episodes, (2) the average, (3) sum, (4) maximum, and (5) minimum duration of the screen-on time. Similar to the location features, we created 6 sets of screen features based on 6 temporal resolutions (ie, 1, 3, 6, 12, 24, and 48 hours preceding each momentary affect report).

##### Time Variables

We included several time variables based on the time of the EMA survey completion as predictors of momentary affect because of emotion’s time-varying nature. We computed one continuous time variable, (1) relative time (relative time in hours since the first EMA survey), and 2 categorical variables: (2) weekend or not (1=yes; 0=no [ie, weekday]) and (3) time of day (midnight to 6 AM: night, 6 AM to noon: morning, noon to 6 PM: afternoon, 6 PM to midnight: evening; dummy coded). Finally, one count variable, (4) prompt (the EMA survey number), was used as the time indicator required by the time-series deep learning algorithm.

### Data Preprocessing

Before imputation, we screened the passive-sensing predictors for near-constant and redundant features: a predictor was dropped if (1) it was near constant, defined as a single value accounted for at least 99% of its nonmissing observations, (2) it was highly correlated with another one, defined as an absolute Spearman correlation over 0.95. We dropped the one with higher missingness in the highly correlated pair. We then handled missing data and extreme values of the smartphone sensor features for each timescale by performing 6 imputations and then 6 winsorizations. For each of the 6 feature sets, we performed single imputation for the missing values in predictors using an iterative imputer (IterativeImputer) that uses the chained equations (MICE [Multivariate Imputation by Chained Equations]) algorithm from the scikit-learn library [39] in Python. Instead of performing imputations for each smartphone sensor (locations, calls, and screen), we performed imputations across smartphone sensor data simultaneously, in which IterativeImputer could use interactions between different sensor data to impute missing values. Importantly, we excluded outcome variables (ie, NA and PA) from imputations to avoid data leakage and overfitting of the prediction models. Finally, we winsorized extreme values in the predictors using the quantile-based method, where we replaced values above the 99th percentile with the 99th-percentile value and replaced values smaller than the 1st percentile with the 1st-percentile value.

To avoid data leakage, the feature screening, feature selection, imputation, and winsorization were all performed in the training set only. The feature list, imputation model, and clipping bounds from the training set were then applied to the validation and test sets. For the TFT, the same principle applied to the participant-level target normalizer used to construct the NA and PA center and scale, which was fit on the training set and reused, rather than refit, in the validation and test sets. A similar training-only-fitted preprocessing pipeline (including standardization for the ElasticNet [elastic net regression]) was also performed for approaches using the ElasticNet and XGBoost (extreme gradient boosting).

### Training and Testing Split

We used a temporally ordered split approach, splitting observations within each individual into three subsets: (1) a test set for final model evaluation, comprising the last 4 consecutive EMA surveys (~1 day); (2) a validation set for hyperparameter tuning, early stopping, and, for model selection between ElasticNet, XGBoost, and N-BEATS (neural basis expansion analysis for interpretable time series forecasting; described below), comprising the 4 consecutive EMA surveys immediately preceding the test window; (3) a training set with all earlier entries (~85% of all EMA surveys). Because the validation and test windows followed the training window, this setup prevents temporal leakage and evaluates short-term within-person generalization. This approach resulted in 6273/6269 EMAs in the training set, 532/531 in the validation set, and 532/532 in the test set for NA and PA, respectively. The training set of 6273 (NA) and 6269 (PA) EMAs allowed 6672 (NA) and 6669 (PA) rolling training sequences (encoder length: 8‐16, prediction length: 1‐4; window lengths were allowed to be flexible and can overlap, so they can exceed observation counts), across 133 participants. The small NA and PA differences reflect outcome-specific missingness. Because the sliding window allows the same EMA to be reused as parts of multiple different-length windows (eg, first window being 1‐11 EMAs predicting 12‐15 EMAs and second window being 2‐13 EMAs predicting 14‐16 EMAs), the number of training sequences to fit the TFT exceeds the number of EMAs in the training set.

For the TFT, we allowed the encoder length to vary between 8 and 16 time points and the prediction length to vary between 1 and 4 time points. As a result, training sequences consist of different combinations of encoder and decoder lengths (eg, 1‐11 EMAs predicting the next 2 EMAs). The final TFT configuration used an encoder length of 16 observations because this window spans roughly 3 days of recent data, given the study’s sampling schedule of approximately 5 EMAs per day. We considered this interval sufficiently long to capture short-term temporal patterns in affect while minimizing data loss that would occur from requiring longer continuous observation histories. A prediction length of 4 was chosen to forecast affect over approximately the subsequent day, directly aligning with the study’s objective of predicting near-term affective dynamics which may ultimately be relevant for timely, adaptive interventions. The final TFT configuration used a maximum encoder length of 16 observations and a maximum prediction length of 4 observations.

### Statistical Analysis

#### Model Evaluations

Model performance metrics were mean absolute error (MAE), root-mean-square error (RMSE), and coefficient of determination (*R*^2^). The MAE is the average of all the absolute values of the differences between the predicted values and the actual values. The RMSE is the square root of the mean of the squared differences between predicted values and actual values. *R*^2^ represents the proportion of variance explained by the model. When *R^2^* equals zero, a model explains no more than the average score. When *R*^2^ equals 1, a model perfectly explains all the outcome variances. Unlike *R*^2^, lower values of MAE and RMSE indicate better performance. Both MAE and RMSE are in the original scale of the outcomes and indicate the extent to which predicted values deviate from the actual values. RMSE is more sensitive to extreme deviations. When MAE or RMSE equals zero, the model perfectly predicts the actual values. When RMSE equals the SD of the outcome variable, the model performs no better than the average score.

#### Person-Mean Affect: For Baseline

In this baseline model, the predicted value for each held-out validation or test observation was set to each participant’s mean NA and PA intensity calculated across the training set. This benchmark required no model fitting or hyperparameter tuning and operated without smartphone sensing or temporal information.

#### ElasticNet and XGBoost Models: For Nonpersonalized Sensing, Personalized Time, and Personalized Sensing Approaches

ElasticNet regression [40], implemented in scikit-learn, and XGBoost (gradient-boosted trees) [41] were used to fit three model variants: (1) nonpersonalized sensing model, (2) personalized time (incorporating participant ID and time variables, without smartphone-sensing features), and (3) personalized sensing (incorporating participant ID, time, and smartphone sensing features, without affect history). Both ElasticNet and XGBoost used a standardized preprocessing pipeline (z-scoring of continuous predictors) prior to model fitting. For ElasticNet, we evaluated 21 hyperparameter combinations crossing 7 regularization strengths (α=0.001, 0.003, 0.01, 0.03, 0.1, 0.3, 1.0) with 3 L1 mixing parameters (l1_ratio=0.1, 0.5, 0.9), each fit for up to 20,000 iterations. For XGBoost, we searched 6 candidate configurations varying the number of trees (200-500), maximum tree depth (2-3), learning rate (0.02‐0.05), row and column subsampling (0.8‐1.0), minimum child weight (1-5), and L1/L2 regularization strength. Within each of the 6 sensor aggregation timescales and 2 semantic-location label versions, the hyperparameter configuration with the lowest validation-set RMSE was selected. Across test sets, the specific timescale and semantic label specifications that yielded the lowest test-set MAE were selected and reported as the top-performing model under each.

#### N-BEATS Model: For Personalized Affective History Approach

To evaluate the personalized affect-history approach, we implemented N-BEATS, a neural network for time-series forecasting composed of stacks of fully connected blocks with backward (backcast) and forward (forecast) residual connections. Unlike the TFT, N-BEATS relied exclusively on each participant’s own historical affect sequence as input, incorporating no smartphone-sensor features. We used fixed-length sequence windows of 16 encoder time points and 4 prediction time points to match the TFT’s maximum window. Outcomes were normalized within participants using a group normalizer with a softplus transformation, and missing time steps were permitted. We set the N-BEATS stack widths to 32 and 64 and the backcast loss ratio to 0, thereby weighting the training loss entirely toward the forecast; all other architecture parameters retained the PyTorch Forecasting defaults. Training used a learning rate of 0.001, weight decay of 0.01, batch size of 64, and gradient clipping at 0.01. We monitored validation loss and retained the checkpoint with the lowest validation loss. Training stopped after 5 consecutive validation checks without an improvement of at least 0.0001, with a maximum of 30 epochs. Training was deterministic and used a random seed of 42. This fixed-window procedure yielded 3746 (NA) and 3741 (PA) windowed training sequences derived from the same underlying training observations described above.

#### Temporal Fusion Transformer: For Full-Information and Personalized Sensing Approaches

To test hypothesis 1, we compared TFTs’ performance (ie, *R*^2^, MAE, and RMSE) with that reported in existing studies [12,13]. For exploratory aim 1, we examined TFTs’ performance across 6 temporal scales (paired n=133). To test hypotheses 2a and 2b, the distal and proximal effects of different types of smartphone-tracked behaviors, we evaluated the top feature importance weights across timescales across encoder and decoder inputs. Because raw TFT variable importance weights reflect nonlinear transformations, we cross-referenced top predictors with statistically significant effects from within-person linear mixed-effects models (detailed below). We used up to 16 consecutive data points (encoder 8‐16) of smartphone sensor data (past inputs) to predict the following up to 4 time points (decoder 1‐4; future inputs) of momentary affect. The important predictors in the past inputs represent more distal factors, whereas future inputs represent more proximal factors in predicting momentary affect. In addition, important predictors in larger timescales and past inputs indicate features with more distal effects, whereas those in smaller timescales and future inputs indicate more proximal or immediate features. For hypothesis 3a, which tested whether self-reported semantic-location features changed performance, we used the paired participant-clustered bootstrap and participant-level Wilcoxon procedures described for aim 1. To test hypothesis 3b, we compared the pattern of important past and future inputs before and after incorporating self-report semantic location features.

All TFT models were implemented in the PyTorch Lightning framework through Google Colab Pro (NVIDIA A100-SXM4-40GB, 40 GB RAM). Model configurations were: (1) initialized the learning rate at 0.001 and then determined it via a systematic finder. The learning rate suggested by the systematic finder was scaled down by a factor of 4 in model training; (2) set the hidden size to 64 and hidden continuous size of 32 to deal with complex features; (3) added 4 multihead attention heads to capture multiscale temporal dependencies; (4) applied a dropout rate of 0.1 and gradient norm clipping threshold of 0.1 in the network to avoid overfitting; (5) used the Quantile loss function for probabilistic interval predictions; and (6) set the batch size to 10 to balance memory footprint and gradient estimation stability.

Using the validation set described above, we monitored validation loss after each epoch, retained the checkpoint with the lowest validation loss, and applied early stopping after 5 consecutive epochs without a minimum improvement of 0.0001 (up to 100 epochs). The retained checkpoint was used for all subsequent test-set evaluation. To make personalized predictions, the full-information TFT included 4 static covariates: participant ID, sequence encoder length, NA or PA center (individual’s mean affect intensities), and NA or PA scale (individual SD of affect intensities). To isolate variance sources for our aim 1, we specified a separate personalized sensing TFT model by refitting the optimal specification for NA and PA after omitting static affect-history statistics (NA or PA center and NA or PA scale). Test-set performance for this model was compared against the ElasticNet and XGBoost baselines to select the best models under the personalized sensing approach.

#### CIs and Significance Testing

We computed 95% CIs for *R*^2^*,* RMSE, and MAE using a participant-level bootstrap. To account for the nonindependence of repeated observations within individuals, resampling was performed at the participant level rather than the observation (EMA) level. Specifically, participants (not individual rows) were resampled with replacement for 2000 resamples, and all test-set rows of each selected participant were pooled. Model performance (*R*^2^, RMSE, and MAE) was computed for each resample, and the 2.5th and 97.5th percentiles of the resulting bootstrap distribution were the CI.

To test aim 1, which was to evaluate whether each approach’s held-out predictive performance differed from the baseline (person-mean affect), we conducted 2 complementary paired statistical tests applied to each approach’s best-performing specification (see above). First, we performed a primary bootstrapping comparison by calculating the MAE difference (ΔMAE=MAE_Approach_ – MAE_Baseline_) for each EMA in the test set. Using 2000 resamples at the participant level (with a fixed random seed of 42 for reproducibility), we derived a 95% CI and a 2-sided bootstrap *P* value (defined as twice the smaller proportion of resampled ΔMAE values falling at or below zero vs at or above zero). Second, as a supplementary test, we conducted a participant-level paired Wilcoxon signed-rank test comparing paired series of mean per-participant MAE_Approach_ and MAE_Baseline_ values, avoiding the assumption of independent repeated observations. To remain conservative, an approach’s improvement over the baseline was considered statistically significant only when both tests met the significance threshold of α=.05. Effect sizes for these paired comparisons were reported as Cohen *d*_z_ (mean of participant-level ΔMAE divided by its SD, accompanied by approximate 95% CIs).

For exploratory aim 1 (evaluating whether TFT performance differed across the 6 temporal aggregation windows), we conducted a nonparametric Friedman test on a participant-by-window matrix containing each participant’s mean MAE at each timescale (n=133). When the omnibus test indicated significant differences across windows, we conducted a post hoc analysis using all 15 pairwise Wilcoxon signed-rank tests between timescales, adjusting for multiple comparisons using the Benjamini-Hochberg false discovery rate (FDR) procedure. For hypothesis 3a (testing whether incorporating self-reported semantic-location features improved model performance), we applied the same dual-testing procedure as in aim 1, a paired participant-clustered bootstrap alongside a participant-level paired Wilcoxon signed-rank procedure.

#### Linear Mixed-Effect Models

Since the TFTs extract the importance of a feature but not the direction or the magnitude of associations, we further examined the within-person associations with a series of linear mixed-effects models. Models were estimated using restricted maximum likelihood (REML) via the lmer() function in the *lme4* package in R (R Core Team; accessed via pymer4 in Python). All variables were standardized (z-scored) before analysis to yield standardized coefficients β. All predictors were person-mean centered to only investigate within-individual associations. In each linear mixed-effect model, momentary NA or PA intensities were outcomes and were regressed on one smartphone-tracked predictor (eg, person-mean-centered total distance traveled in the 3 hours preceding the survey). In each model, we included a random intercept to handle between-person baseline differences in affect and included a random slope to allow the relationship between the predictor and outcome to vary across individuals.

## Results

### Participants Characteristics

The final analytic sample consisted of 133 adults (mean age 36.4, SD 12.0, age range: 19‐63 years). Participants’ gender composition was as follows: 71 out of 133 (53.4%) women, 51 out of 133 (38.3%) men, 3 out of 133 (2.3%) gender diverse, and 8 out of 133 (4.9%) unknown. Their race was 91 out of 133 (68.4%) White, 14 out of 133 (11.8%) Asian, 12 out of 133 (6.9%) Black or African American, 7 out of 133 (5.3%) others, and 9 out of 133 (6.8%) preferred not to say. A total of 9 out of 133 (6.8%) of the analytic sample reported being Latinx or Hispanic.

### Aim 1: Model Performance in Predicting Momentary NA and PA

Table 2 presents the best-performing model under different approaches (ie, including different sets of predictors and algorithms). Results showed that models that simply used the mean affect intensity for each person (predictors: ID + training NA or PA) showed moderate performance on unseen test sets (NA: *R*²=40.2% [95% CI 31-48.2], RMSE=0.89 [95% CI 0.80-0.97], MAE=0.66 [95% CI 0.60-0.73]); PA: *R*²=36.1% [95% CI 20.4-48.3], RMSE=0.93 [95% CI 0.84-1.03], MAE=0.71 [95% CI 0.65-0.78]). Nonpersonalized sensing models performed significantly worse than the baseline for both outcomes (both *P* values<.001; NA Cohen *d*=0.56; PA Cohen *d*=0.37). Among personalized benchmarks, the ID-and-time ElasticNet was significantly worse for NA (ΔMAE=0.020, 95% CI 0.004-0.036; *P*_bootstrap_=.01; *P*_Wilcoxon_=.04; Cohen *d*=0.21), whereas the affect-history N-BEATS model did not differ for NA. N-BEATS produced a statistically significant but very small improvement for PA (ΔMAE=−0.002, 95% CI −0.003 to−0.000; *P*_bootstrap_=.04; *P*_Wilcoxon_=.01; Cohen *d*=−0.18). The personalized sensing TFT without Affect Center and Scale did not differ from the baseline for either outcome. The full-information TFT significantly improved NA prediction (ΔMAE=−0.040, 95% CI −0.065 to−0.015; *P*_bootstrap_=.001; *P*_Wilcoxon_=.004; Cohen *d*=−0.27) but not PA prediction (ΔMAE=−0.006, 95% CI −0.031 to 0.020; *P*_bootstrap_=.66; *P*_Wilcoxon_=.13; Cohen *d*=−0.04). The best full-information models used 6-hour features with self-reported semantic locations for NA and 1-hour features with self-reported semantic locations for PA.

**Table 2.** Prediction performances of different model approaches when predicting momentary NA^a^ and PA^b^ on the test dataset (n=133 individuals; total 532 surveys)^c^.

Approach	Model	Feature sets	*R*² (%) (95% CI)	RMSE^d^ (95% CI)	MAE^e^ (95% CI)	ΔMAE vs baseline (95% CI)	*P* value (bootstrap)	*P* value (Wilcoxon)	Cohen *d*
NA									
Person-mean NA	Mean training NA	No passive sensing	40.2 (31.0 to 48.2)	0.89 (0.80 to 0.97)	0.66 (0.60 to 0.73)	—^f^	—	—	—
Nonpersonalized sensing	XGBoost^g^	48h+OSM^h^	4.6 (−2.3 to 10.5)	1.12 (1.01 to 1.23)	0.89 (0.80 to 0.97)	0.222 (0.158 to 0.295)	<.001	<.001	0.56
Personalized time	ElasticNet^i^	No passive sensing	38.2 (29.6 to 45.6)	0.90 (0.81 to 0.99)	0.68 (0.62 to 0.75)	0.020 (0.004 to 0.036)	.01	.04	0.21
Personalized sensing	TFT^j^	6h+OSM+self-report locations	36.6 (26.3 to 45.8)	0.91 (0.82 to 1.01)	0.66 (0.59 to 0.73)	−0.002 (−0.020 to 0.018)	.86	.89	−0.01
Personalized NA history	N-BEATS^k^	No passive sensing	35.5 (25.3 to 44.5)	0.92 (0.82 to 1.02)	0.66 (0.59 to 0.74)	−0.001 (−0.022 to 0.019)	.93	.88	−0.01
Full-information	TFT	6h+OSM+self-report locations	45.2 (33.8 to 54.3)	0.85 (0.76 to 0.94)	0.62 (0.56 to 0.69)	−0.040 (−0.065 to −0.015)	.001	.004	−0.27
PA									
Person-mean PA	Mean training PA	No passive sensing	36.1 (20.4 to 48.3)	0.93 (0.84 to 1.03)	0.71 (0.65 to 0.78)	—	—	—	—
Nonpersonalized sensing	XGBoost	48h+OSM+self-report locations	10.8 (1.4 to 18.7)	1.10 (1.01 to 1.19)	0.87 (0.80 to 0.95)	0.163 (0.091 to 0.239)	<.001	<.001	0.37
Personalized time	ElasticNet	No passive sensing	35.8 (20.5 to 47.2)	0.93 (0.85 to 1.03)	0.72 (0.66 to 0.79)	0.009 (−0.008 to 0.028)	.34	.46	0.09
Personalized sensing	TFT	1h+OSM+self-report locations	34.8 (17.7 to 48.2)	0.94 (0.84 to 1.05)	0.70 (0.64 to 0.78)	−0.007 (−0.029 to 0.016)	.59	.21	−0.05
Personalized PA history	N-BEATS	No passive sensing	35.4 (20.2 to 48.1)	0.93 (0.83 to 1.03)	0.71 (0.65 to 0.77)	−0.002 (−0.003 to −0.000)	.04	.01	−0.18
Full-information	TFT	1h+OSM+self-report locations	32.5 (11.6 to 47.8)	0.96 (0.84 to 1.08)	0.70 (0.63 to 0.78)	−0.006 (−0.031 to 0.020)	.66	.13	−0.04

a NA: negative affect.

b PA: positive affect.

c *R*², RMSE, and MAE are computed on the held-out test set; brackets denote the 95% participant-level bootstrap CI (2000 resamples with replacement). ΔMAE vs Baseline=MAE_approach_- MAE_Person-Mean Baseline_, calculated from a paired participant-level bootstrap (positive values indicate worse performance than baseline, negative values indicate improvement). The *P* (bootstrap) value represents the 2-sided bootstrap *P* value for this difference; *P* value (Wilcoxon) is derived from a participant-level paired Wilcoxon signed-rank test on MAE (n=133 participants rather than 532 observations, to avoid pseudoreplication). Cohen *d* indicates the paired-samples effect size (mean difference divided by the SD of the MAE differences). Bold text indicates a statistically significant improvement or decline relative to the person-mean baseline (requiring both bootstrap and Wilcoxon *P*<.05). For approaches incorporating passive sensing feature sets (combinations of time windows and semantic-location label sets), the row displays the best-performing specification based on test-set MAE.

d RMSE: root-mean-square error.

e MAE: mean absolute error.

f Not applicable.

g XGBoost: extreme gradient boosting.

h OSM: OpenStreetMap.

i ElasticNet: elastic net regression.

j TFT: temporal fusion transformer.

k N-BEATS: neural basis expansion analysis for interpretable time series forecasting.

### Aim 2: Role of Timescales in Model Performance and Variable Importance

#### Overview

Figure 2 shows MAE across the 6 aggregation windows. In the figure, shaded bands represent 95% participant-level bootstrap CIs. In-panel statistics report the Friedman omnibus test across all 6 time windows and the range of FDR-corrected pairwise Wilcoxon *P* values across all 15 window-pair comparisons for each semantic-location feature set. For NA, neither the OpenStreetMap-only specification (*χ*²_5_=5.93, *P*=.31) nor the OpenStreetMap-plus-self-report specification (*χ*²_5_=10.10, *P*=.07) differed significantly across windows. FDR-corrected pairwise *P* values ranged from .05 to .98. For PA, the OpenStreetMap-only omnibus test was nonsignificant (*χ*²_5_=2.16, *P*=.83), whereas the OpenStreetMap-plus-self-report omnibus test reached significance (*χ*²_5_=11.21, *P*=.05), which was driven by the worst performance using the 48-hour OpenStreetMap-plus-self-report specification. However, none of the PA pairwise comparisons survived FDR correction (*P*_FDR_=.08-.99). Thus, no specific pair of aggregation windows showed a robust performance difference.

#### Variable Importance Across Timescales

**Figure 2.**
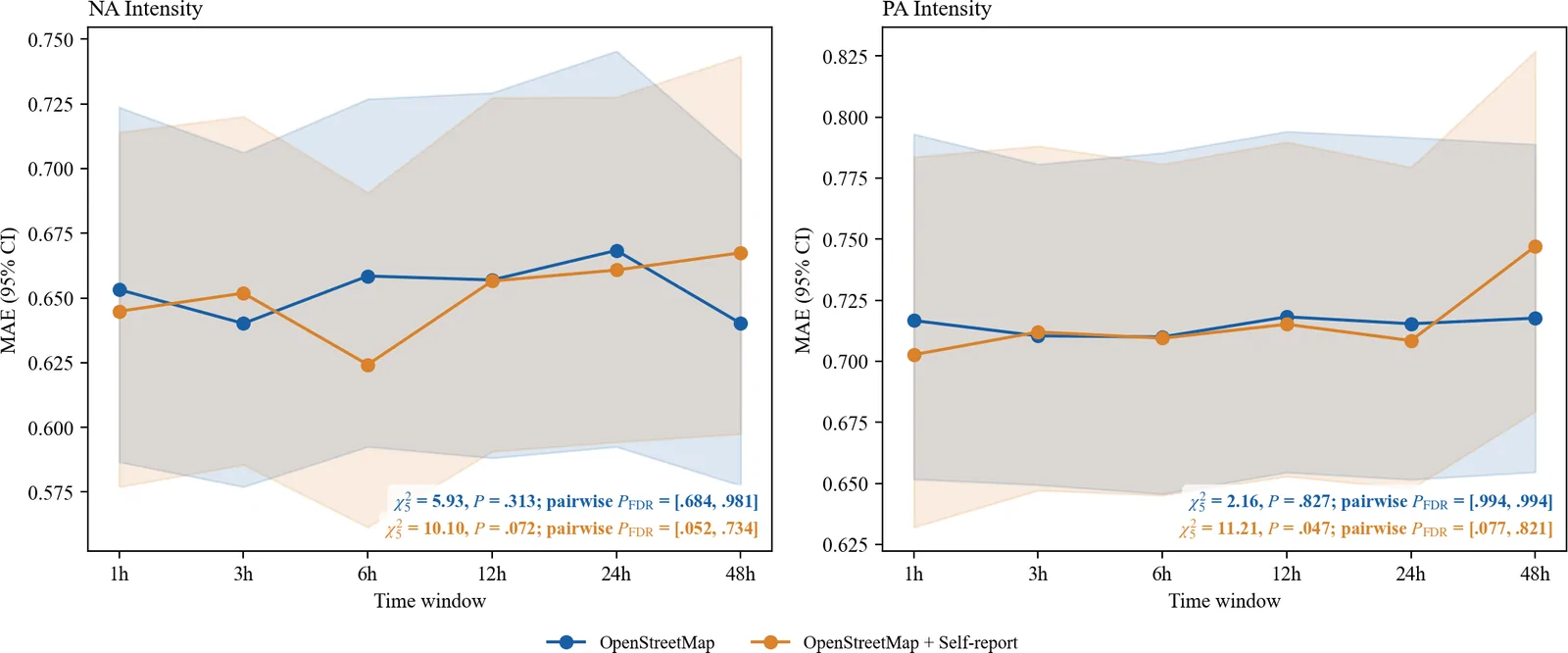
Temporal fusion transformer (TFT) mean absolute error (MAE) across time windows (1, 3, 6, 12, 24, and 48 hours) in predicting momentary negative affect (NA, left) and positive affect (PA, right) intensity, for semantic-location features derived from OpenStreetMap versus OpenStreetMap supplemented with self-report in the test set (n=133 individuals; total 532 surveys). FDR: false discovery rate.

Figure 3 presents a heatmap of important past (left) versus future inputs (right) identified by the TFTs across time windows for predicting NA (upper 2 panels) and PA (lower 2 panels) using location features from OpenStreetMap (blue names) versus from OpenStreetMap supplemented with self-report. We did not find consistent support for hypothesis 2a. Although behavioral indicators of social interactions (ie, calls) and screen interactions appeared among the important future inputs for both NA and PA, this holds only before supplementing OpenStreetMap with self-reported locations to refine location features. After supplementing OpenStreetMap with self-reported locations to refine location features, percent time at leisure places (self-report) was an important future input for both NA and PA, especially in the 3-hour window (NA 0.16, PA 0.17). Partially supporting hypothesis 2b, partial-mobility features contributed as both past and future inputs, with number of location transitions and variance in speed as notable proximal (future) inputs and radius of gyration and number of significant places as distal (past) inputs. Not supporting hypotheses 2a and 2b, there is no clear pattern in variable importance across time windows (eg, consistently darker or lighter from left to right).

**Figure 3.**
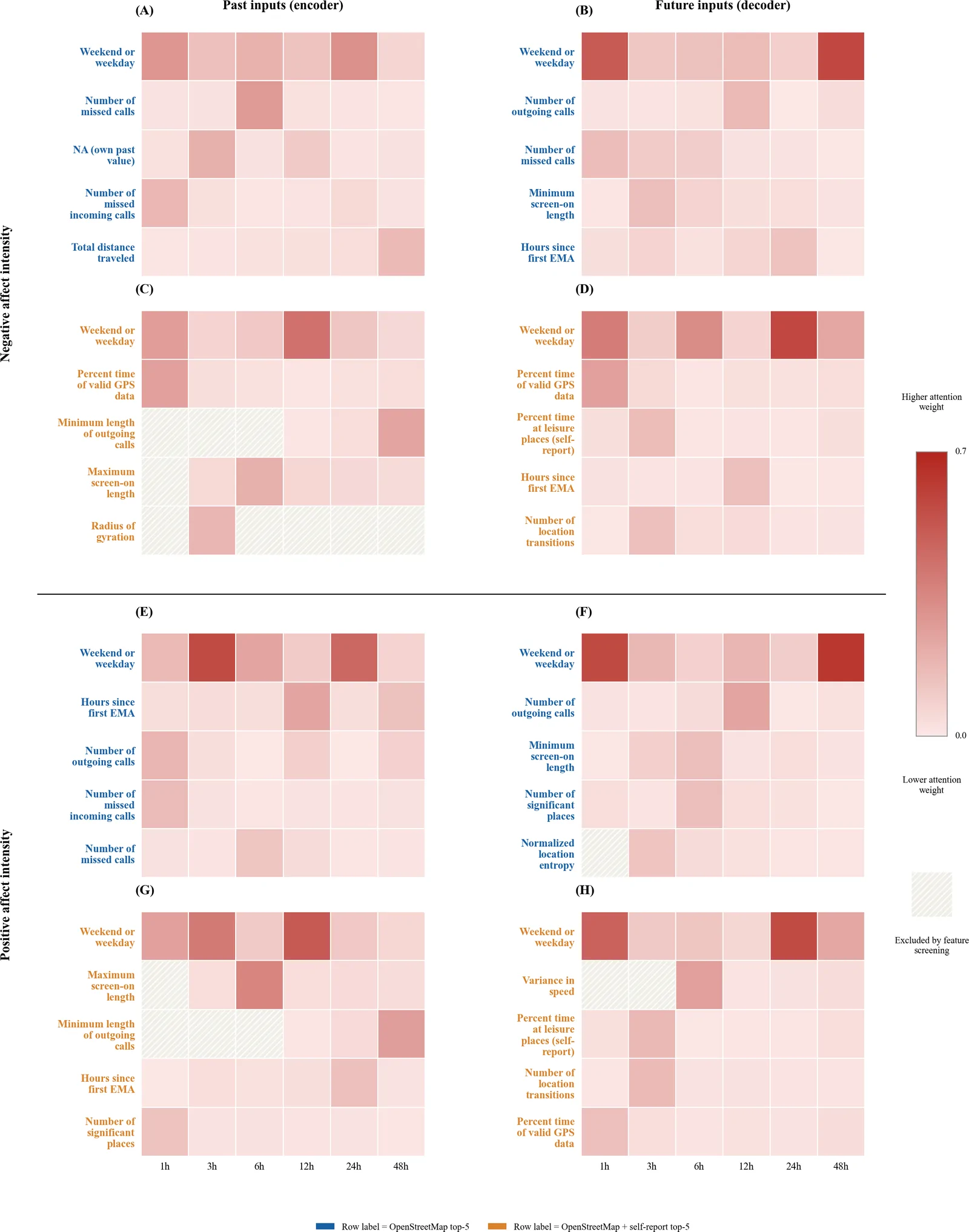
Heatmap of important past (left, panels A, C, E, and G) versus future inputs (right, panels B, D, F, and H) identified by temporal fusion transformer (TFT) across time windows for predicting negative affect (NA; upper panels A-D) and positive affect (PA; lower panels E-H) using location features from OpenStreetMap (blue names) versus from OpenStreetMap supplemented with self-report. EMA: ecological momentary assessment.

Outside of hypotheses, the individual’s affect history was an important predictor across time windows for both NA and PA (left panels in Figure 3). Across time windows, a time variable (weekend or weekday) is an important future input and, sometimes, a past input across location feature sets for NA and PA, especially at the longer windows (24-hour as a future input and 12-hour as a past input; Figure 3).

### Aim 3: The Role of Self-Report Semantic Locations

#### Overview

Overall, both bootstrapping and Wilcoxon significance tests suggested that adding self-report semantic-location features did not significantly change prediction accuracy for either NA or PA. Specifically, for predicting NA intensity, the best TFT using semantic-location features derived from OpenStreetMap only, which was based on 3-hour features (MAE=0.64), did not perform significantly differently from the TFT using semantic-location features from OpenStreetMap supplemented with self-report locations, which was based on 6-hour features (MAE=0.62; ΔMAE [95% CI]=0.016 [−0.009 to 0.040]; *P*_bootstrap_=0.21; *P*_Wilcoxon_=0.35; Cohen *d* [95% CI]=0.11 [−0.06 to 0.28]). Results were similar for PA, such that the best TFT before incorporating self-report locations, which was based on 6-hour features (MAE=0.71), did not perform significantly differently from the best TFT after incorporating self-report locations, which was based on 1-hour features (MAE=0.70; ΔMAE [95% CI]=0.007 [−0.012 to 0.026]; *P*_bootstrap_=0.44; *P*_Wilcoxon_=0.34; Cohen *d* [95% CI]=0.07 [−0.10 to 0.24]).

#### Variable Importance After Adding Self-Report Semantic Locations

Figure 4 presents the attention weights of past (left) versus future inputs (right) from the best-performing TFT for NA intensity (top) and PA intensity (bottom). Blue bars represent attention weights before adding self-report semantic locations, whereas orange bars represent attention weights after adding self-report semantic locations. Consistent with hypothesis 3b, incorporating self-report semantic locations changed the composition and relative ranking of important inputs, although the pattern of change varied across past and future inputs. The percent time spent at leisure places (self-report) emerged as an important input in the TFT attention weights, particularly at the 3-hour window for both NA and PA (Figure 3; NA: 0.16, PA: 0.17; see detailed attention weights for each variable in Tables S1 and S2 in Multimedia Appendix 1). Several GPS features indicating spatial mobility (variance in speed, number of location transitions, and radius of gyration) were identified among important inputs across models.

**Figure 4.**
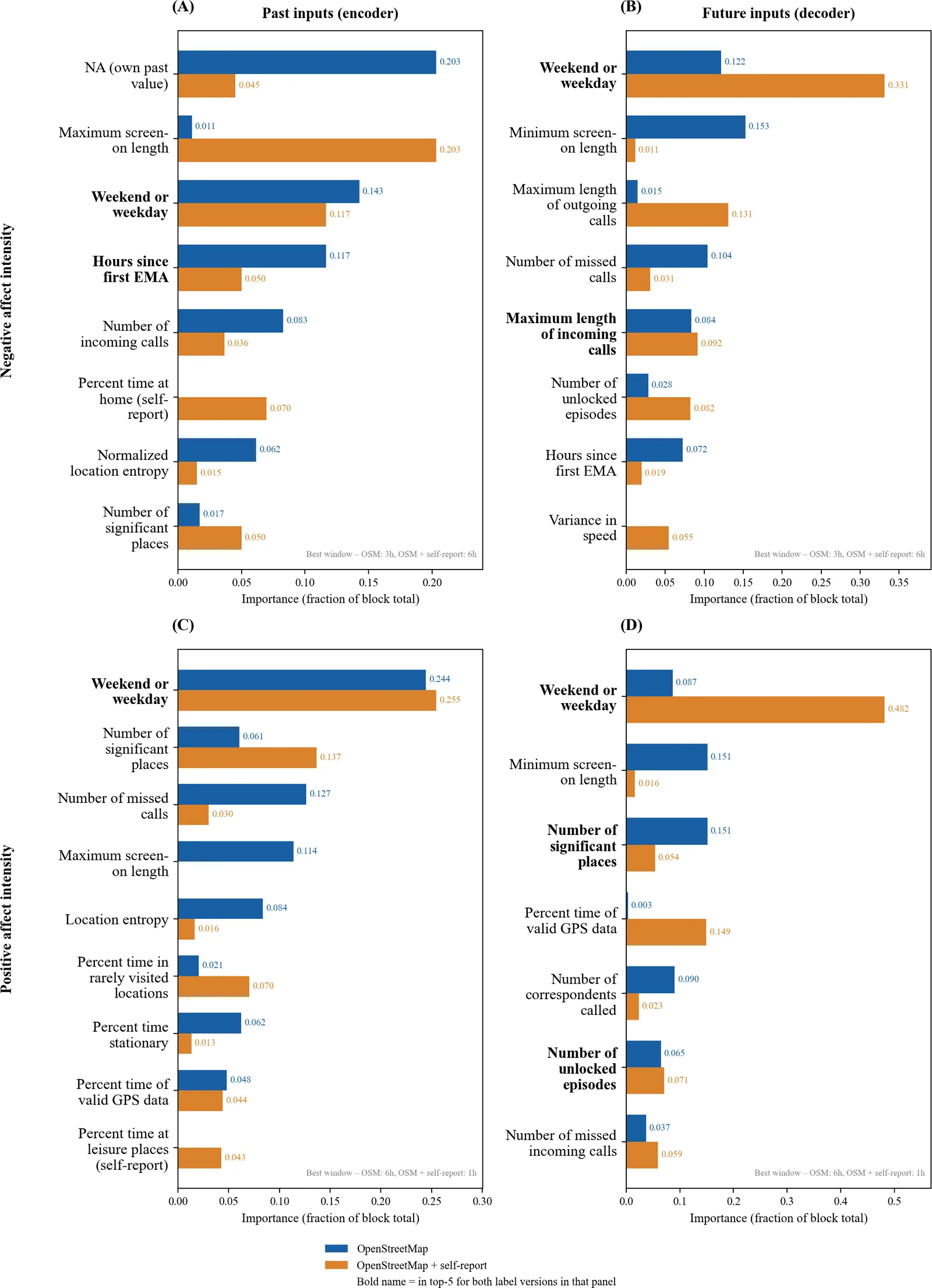
Attention weights of top-5 past (left; panels A and C) versus future inputs (right; panels B and D) from the best-performing temporal fusion transformer (TFT) in predicting momentary negative affect (NA, top, panels A and B) and positive affect (PA, bottom, panels C and D) intensity, for semantic-location features derived from OpenStreetMap (blue) versus OpenStreetMap supplemented with self-report (orange) in the test set (n=133 individuals; total 532 surveys). EMA: ecological momentary assessment.

### Within-Individual Associations in Linear Mixed-Effects Models

Tables S1 and S2 present standardized coefficients in linear mixed-effects models for NA and PA, respectively (see them in Multimedia Appendix 1). Smartphone-tracked behaviors showed weak, if any, bivariate associations with momentary affect intensity (all |β|<0.06). After FDR correction across 236 tests per outcome, 5 proximal call features were positively associated with NA: the numbers of incoming calls, outgoing calls, and correspondents in the 1-hour window and the numbers of outgoing calls and correspondents in the 3-hour window (β=0.03-0.06, *P*_FDR_=.003-.05). Thirteen features were associated with PA after correction. PA was positively associated with location diversity across 3- to 24-hour windows (log location variance, location entropy, normalized location entropy, and number of significant places; β=0.04-0.06) and negatively associated with time at self-reported workplaces in the 1-, 3-, and 6-hour windows and with the number of correspondents in the 1-hour window (β=–0.04 to –0.05; *P*_FDR_=.002-.03).

### Sensitivity Analyses

Three sensitivity analyses supported the robustness of the primary findings. First, lowering the minimum number of valid EMA reports from 40 to 20 retained 167 participants (n=8398 NA EMAs and n=8391 PA EMAs), compared with 133 participants in the primary sample (n=7337 NA EMAs and n=7332 PA EMAs). Predictive performance did not differ significantly for either NA (6-hour, OSM + self-report; ΔMAE=0.04, 95% CI −0.10 to 0.20; *P*_bootstrap_=.624; *P*_Wilcoxon_=.54) or PA (1-hour, OSM + self-report; ΔMAE=0.12, 95% CI −0.07 to 0.36; *P*_bootstrap_=.26; *P*_Wilcoxon_=.32). Second, replacing MICE with median imputation did not significantly change performance for NA (6-hour, OSM + self-report; ΔMAE=0.010, 95% CI −0.001 to 0.021; *P*_bootstrap_=.07; *P*_Wilcoxon_=.21) or PA (1-hour, OSM + self-report; ΔMAE=0.0005, 95% CI −0.0046 to 0.0052; *P*_bootstrap_=.84; *P*_Wilcoxon_=.70). Finally, increasing the maximum encoder length from 16 (minimum 8) to 20 (minimum 12), while retaining a maximum decoder length of 4, did not significantly affect performance for NA (6-hour, OSM + self-report; ΔMAE=0.0008, 95% CI −0.0089 to 0.0106; *P*_bootstrap_=.89, *P*_Wilcoxon_=.86) or PA (1-hour, OSM + self-report; ΔMAE=0.0035, 95% CI −0.0080 to 0.0137; *P*_bootstrap_=.53; *P*_Wilcoxon_=.21).

## Discussion

### Principal Findings

This study parsed the contributions of EMA and smartphone sensing data to personalized predictions of momentary affect. On held-out EMA observations, smartphone sensing within the full-information TFT added statistically significant and modest predictive value for NA but small, nonsignificant value for PA relative to the person-mean baseline. Person-specific mean affect accounted for a substantial share of predictive performance. Although one PA omnibus test differed across aggregation windows, no pairwise time-window comparison survived FDR correction. Adding self-reported semantic locations did not significantly improve NA or PA prediction. Before these locations were added, mobility markers and smartphone social interactions appeared among important past and future inputs. After they were added, mobility markers appeared more often as past inputs, partially supporting hypothesis 2b, whereas screen and smartphone social interactions showed no consistent proximal or distal pattern, providing no support for hypothesis 2a. Overall, our results suggest modest added value of smartphone sensing over EMA for NA prediction following person-specific calibration.

### Aim 1: Model Performance in Predicting Momentary NA and PA

Aim 1 tested how much each data source contributed to personalized prediction and whether the TFT improved on a person-mean baseline. Partially consistent with hypothesis 1, smartphone sensing added significant predictive value for NA (but not PA), empowered by transformer-based models. All prior studies made personalized predictions through incorporating all data sources, including both active and passive inputs [11-13]. Ultimately, the primary promise of digital phenotyping is the possibility of generating accurate predictions of momentary affect intensity using passive inputs alone. This is a goal that has attracted increasing interest given its broad clinical and practical implications. The present findings can be situated within a broader affect-dynamics framework in which momentary affect reflects a combination of relatively stable person-specific tendencies, recent affective trajectories, and immediate contextual inputs. Findings showed that participants’ mean affect and affective history accounted for a substantial proportion of predictive performance, whereas smartphone sensing provided only modest incremental information. This pattern indicates that smartphone sensing should not yet be viewed as a standalone replacement for active self-report, but rather as a complementary source of information to EMA. Future personalized prediction research should consistently evaluate the person-mean affect intensity as an essential benchmark and explore richer multimodal passive signals that capture dimensions of emotional experience beyond smartphone-tracked behaviors (eg, physiological measures and linguistic features).

Another motivation of the study was to address the relative neglect of PA in the affective computing literature, particularly given its established importance for psychological well-being and depression treatment. Our findings show that the incremental value of smartphone sensing was significant and modest for NA, but small and nonsignificant for PA. Consequently, high predictive performance for NA (or depressed mood, which is more commonly investigated) should not be assumed to automatically generalize to PA. Current sensing modalities may be differentially informative across valence dimensions. Improving PA prediction may require larger sample sizes or alternative digital markers. Future affective computing research focused on PA is clearly warranted.

### Aims 2 and 3: The Role of Timescales and Self-Reported Semantic Locations

Aims 2 and 3 examined aggregation timescales and self-reported semantic locations. Across 6 windows, 3 omnibus tests were nonsignificant and 1 PA test reached statistical significance, but no pairwise comparison survived FDR correction. Aggregation-window choices may therefore be guided partly by practical considerations, such as computational efficiency, pending replication. Adding self-reported semantic locations did not improve predictive performance, so the value of collecting these data appears to lie more in contextual interpretation than in accuracy.

Three broader patterns emerged from the attention-weight analyses. First, incorporating self-reported semantic locations changed the composition and relative ranking of important inputs despite not improving predictive accuracy, suggesting that contextual information may contribute more to interpretation than to predictive accuracy. These changes did not follow a clear pattern across NA and PA or across past and future inputs. Time at self-reported leisure places appeared as an important input after semantic-location features were added. Second, the TFT prioritized different classes of smartphone-tracked behavior across aggregation windows while maintaining broadly comparable predictions. Third, spatial-mobility features appeared more consistently as past than future inputs, suggesting that they may summarize accumulated behavioral context rather than immediate affective triggers. However, these attention weights indicate model-based predictive relevance, not causal effects, and should be treated more as hypothesis-generating.

### Implications for Research and Applications

The affective computing approach to personalized monitoring of affect intensity is still in its infancy, and this study addressed three key methodological questions: (1) how much variance did smartphone sensing add to personalized prediction of momentary affect beyond EMA baselines, (2) does the data aggregation timescale impact predictive performance, and (3) what is the value of integrating self-report semantic locations. First, our framework provides a more rigorous benchmark for evaluating passive sensing for affect monitoring. Future work on personalized predictions should benchmark models against person-specific mean affect, demonstrating incremental predictive value rather than relying solely on absolute accuracy metrics. Second, because predictive performance remained stable across the 6 evaluated timescales, feature engineering decisions regarding aggregation windows can be guided by practical considerations, such as computational efficiency. Third, incorporating contextual self-reports may enhance the interpretability of digital behavioral markers, even when they do not directly improve prediction accuracy. Finally, rather than replacing EMA, passive sensing may reduce the frequency of active assessment while maintaining continuous, individualized monitoring between self-reports.

### Limitations and Future Directions

This study has several limitations. First, within-person reliability was modest for NA and PA (*ω*=0.64 and 0.69), which constrains achievable predictive accuracy. Future studies should balance more reliable measurement of momentary affect against EMA burden. Second, the feature-importance rankings produced by the TFT reflect associational rather than causal relationships. A predictor may receive high attention weight simply because it is correlated with an unobserved driver or acts as a proxy that reduces prediction error. Consequently, these findings should be interpreted as hypotheses regarding potential mechanisms rather than definitive evidence of causal influence. Experimental manipulation or microrandomized intervention studies are necessary to establish causal pathways.

In addition, our models were good at predicting momentary affect intensity at time points immediately following the training period (about 2 weeks) for the same individuals. However, it is unclear whether our models can make good predictions days, weeks, or even months after the training data time points. Future work could examine whether algorithms can stop assessing self-report experiences after a certain period and then allow ubiquitous and continuous monitoring. If so, it would also be important to clarify the maximum length of the no-report period.

The present findings illustrate AI’s potential to predict momentary affect in daily life (situational generalizability) by forecasting an individual’s future states from their own past data (within-person temporal generalizability). A critical remaining question concerns population generalizability and whether such models can generalize to entirely new individuals. We maintain that, at least for predicting momentary affect intensity, developing personalized models is more feasible than building universal models expected to generalize across individuals. In human-to-human interactions, people can readily recognize distinct emotions in a stranger, but accurately gauging another person’s emotional intensity typically requires familiarity. Consistent with this distinction, research evaluating whether passive sensor data could classify discrete momentary affect states (eg, angry or not angry) across new individuals found that traditional ML models performed only slightly above chance [8]. Although population-level prediction of affect intensity without a priori calibration remains challenging, achieving it would carry substantial public health implications.

Third, the present work is limited by the number of modalities in data sources. Additional smartphone sensors collecting other social interaction data (eg, text messages or social media) and audio information (eg, volumes and frequency of meaningful conversations around an individual) could add modalities while maintaining the benefits from the cheap and wide access of smartphones. Future affect computing research could also benefit from a larger dataset with more people and more observations, as other AI fields (eg, large language models) have illustrated that “a ‘dumb’ algorithm with lots and lots of data beats a ‘clever’ one with modest amounts of data” [42].

### Conclusion

This study highlights the incremental value of smartphone sensing for personalized, short-term prediction of momentary affect beyond simple person-specific affective baselines. Notably, this added predictive value was significant only for NA, while participants’ mean baseline affect accounted for a substantial proportion of overall predictive performance across all models. No pairwise performance difference emerged across the 6 passive-data aggregation windows. Furthermore, incorporating self-reported semantic locations did not improve prediction accuracy, though it provided more interpretable contextual features for generating hypotheses about affect-behavior relations. Overall, these findings suggest that smartphone sensing is currently better positioned to augment rather than replace EMA. Following an initial period of person-specific calibration, passive sensing may estimate short-term affective fluctuations between self-report prompts and support lower-burden or adaptive EMA designs. Replication in larger, more diverse, and clinical samples remains essential before deploying these models in real-world assessment or intervention use.

## Supplementary material

10.2196/90970 Multimedia Appendix 1 Affect distributions, important predictors across temporal and semantic-location feature sets, and prediction performance for discrete affect states.

## Abbreviations

DBSCAN: Density-Based Spatial Clustering of Applications with Noise
ElasticNet: elastic net regression
EMA: ecological momentary assessment
FDR: false discovery rate
MAE: mean absolute error
MICE: Multivariate Imputation by Chained Equations
ML: machine learning
N-BEATS: neural basis expansion analysis for interpretable time series forecasting
NA: negative affect
PA: positive affect
REML: restricted maximum likelihood
RMSE: root-mean-square error
TFT: temporal fusion transformer
XGBoost: extreme gradient boosting
