# Supplementary material for: Incremental Value of Smartphone Sensing for Monitoring Momentary Affect Intensity in Adults Using Transformer-Based Models: Observational Study
Source: JMIR Mhealth Uhealth. 2026 Sep 28;14:e90970. doi: 10.2196/90970 (PMC13626072; doi:10.2196/90970)
Supplement: Supplementary file 1 [file mhealth-v14-e90970-s001.docx]

# Supplemental Materials

**Figure S1.** Survey-level (N = 7,337 NA and 7,332 PA) and individual-level (n = 133) distributions of momentary negative affect (NA) and positive affect (PA) in the final analytic sample.

**Table S1.** The most important variables for temporal fusion transformers to predict momentary negative affect (NA).

| **Momentary NA Intensity**  **With Semantic Location Features**  **From OpenStreetMap** | | | |  | **Momentary NA Intensity**  **With Self-report Semantic Location Features** | | | |
| --- | --- | --- | --- | --- | --- | --- | --- | --- |
| **1-hour interval** | **Importance** | **β** | **p** |  | **1-hour interval** | **Importance** | **β** | **p** |
| **Past Inputs** |  |  |  |  | **Past Inputs** |  |  |  |
| **Weekend or Weekday** | **0.30** | **-0.04** | **0.01** |  | **Weekend or Weekday** | **0.27** | **-0.04** | **0.01** |
| **N of Missed Incoming Calls** | **0.18** | **0.02** | **0.06** |  | **Percent Time of Valid GPS Data** | **0.26** | **0.00** | **0.98** |
| **N of Outgoing Calls** | **0.15** | **0.05** | **<0.001*** |  | N of Significant Places | 0.06 | 0.00 | 0.64 |
| Percent Time at Home (OSM) | 0.06 | 0.01 | 0.36 |  | Percent Time in Rarely Visited Locations | 0.05 | 0.01 | 0.33 |
| **N of Incoming Calls** | **0.06** | **0.03** | **<0.001*** |  | **N of Incoming Calls** | **0.04** | **0.03** | **<0.001*** |
| **Future Inputs** |  |  |  |  | **Future Inputs** |  |  |  |
| **Weekend or Weekday** | **0.51** | **-0.04** | **0.01** |  | **Weekend or Weekday** | **0.39** | **-0.04** | **0.01** |
| **N of Missed Calls** | **0.15** | **0.02** | **0.04** |  | **Percent Time of Valid GPS Data** | **0.26** | **0.00** | **0.98** |
| Percent Time at Home (OSM) | 0.05 | 0.01 | 0.36 |  | **N of Missed Calls** | **0.08** | **0.02** | **0.04** |
| N of Missed Incoming Calls | 0.05 | 0.02 | 0.06 |  | **N of Unlocked Episodes** | **0.05** | **0.02** | **0.03** |
| Max Length of Outgoing Calls | 0.05 | 0.02 | 0.09 |  | N of Missed Incoming Calls | 0.05 | 0.02 | 0.06 |
| **Static Inputs** |  |  |  |  | **Static Inputs** |  |  |  |
| **NA Scale** | **0.67** | **\** | **\** |  | **NA Scale** | **0.60** | **\** | **\** |
| **Participant ID** | **0.32** | **\** | **\** |  | **Participant ID** | **0.36** | **\** | **\** |
| **NA Center** | **0.27** | **\** | **\** |  | **NA Center** | **0.29** | **\** | **\** |
| Encoder Length | 0.07 | \ | \ |  | Encoder Length | 0.09 | \ | \ |
| **3-hour interval** | **Importance** | **β** | **p** |  | **3-hour interval** | **Importance** | **β** | **p** |
| **Past Inputs** |  |  |  |  | **Past Inputs** |  |  |  |
| **NA (Own Past Value)** | **0.20** | **\** | **\** |  | **Radius of Gyration** | **0.18** | **0.00** | **0.71** |
| **Weekend or Weekday** | **0.14** | **-0.04** | **0.01** |  | **N of Correspondents Called** | **0.15** | **0.05** | **<0.001*** |
| **Hours Since EMA1** | **0.12** | **0.02** | **0.22** |  | N of Missed Outgoing Calls | 0.08 | 0.02 | 0.08 |
| **N of Incoming Calls** | **0.08** | **0.04** | **0.004** |  | **Weekend or Weekday** | **0.08** | **-0.04** | **0.01** |
| Normalized Location Entropy | 0.06 | -0.01 | 0.34 |  | Percent Time at Leisure Places (Self-report) | 0.06 | 0.00 | 0.84 |
| **Future Inputs** |  |  |  |  | **Future Inputs** |  |  |  |
| **Min Screen-on Length** | **0.15** | **0.00** | **0.84** |  | **Percent Time at Leisure Places (Self-report)** | **0.16** | **0.00** | **0.84** |
| **Weekend or Weekday** | **0.12** | **-0.04** | **0.01** |  | **N of Location Transitions** | **0.14** | **0.00** | **0.89** |
| **N of Missed Calls** | **0.10** | **0.03** | **0.04** |  | **Weekend or Weekday** | **0.10** | **-0.04** | **0.01** |
| **Max Length of Incoming Calls** | **0.08** | **0.02** | **0.03** |  | Normalized Location Entropy | 0.08 | -0.01 | 0.34 |
| Hours Since EMA1 | 0.07 | 0.02 | 0.22 |  | **N of Outgoing Calls** | **0.07** | **0.05** | **<0.001*** |
| **Static Inputs** |  |  |  |  | **Static Inputs** |  |  |  |
| **NA Scale** | **0.58** | **\** | **\** |  | **NA Scale** | **0.62** | **\** | **\** |
| **NA Center** | **0.32** | **\** | **\** |  | **NA Center** | **0.32** | **\** | **\** |
| **Participant ID** | **0.31** | **\** | **\** |  | **Participant ID** | **0.30** | **\** | **\** |
| **Encoder Length** | **0.12** | **\** | **\** |  | Encoder Length | 0.09 | \ | \ |
| **6-hour interval** | **Importance** | **β** | **p** |  | **6-hour interval** | **Importance** | **β** | **p** |
| **Past Inputs** |  |  |  |  | **Past Inputs** |  |  |  |
| **N of Missed Calls** | **0.28** | **0.01** | **0.35** |  | **Max Screen-on Length** | **0.20** | **-0.01** | **0.41** |
| **Weekend or Weekday** | **0.20** | **-0.04** | **0.01** |  | **Weekend or Weekday** | **0.12** | **-0.04** | **0.01** |
| Location Variance | 0.08 | 0.01 | 0.49 |  | Percent Time at Home (Self-report) | 0.07 | 0.00 | 0.89 |
| Percent Time Stationary | 0.08 | -0.01 | 0.50 |  | N of Significant Places | 0.05 | -0.02 | 0.12 |
| **Location Entropy** | **0.07** | **-0.02** | **0.04** |  | Hours Since EMA1 | 0.05 | 0.02 | 0.22 |
| **Future Inputs** |  |  |  |  | **Future Inputs** |  |  |  |
| **Weekend or Weekday** | **0.14** | **-0.04** | **0.01** |  | **Weekend or Weekday** | **0.33** | **-0.04** | **0.01** |
| **Location Entropy** | **0.14** | **-0.02** | **0.04** |  | **Max Length of Outgoing Calls** | **0.13** | **0.01** | **0.45** |
| N of Missed Calls | 0.10 | 0.01 | 0.35 |  | Max Length of Incoming Calls | 0.09 | 0.01 | 0.50 |
| N of Significant Places | 0.09 | -0.02 | 0.12 |  | N of Unlocked Episodes | 0.08 | -0.01 | 0.27 |
| **N of Correspondents Called** | **0.08** | **0.03** | **0.02** |  | Variance in Speed | 0.05 | 0.01 | 0.60 |
| **Static Inputs** |  |  |  |  | **Static Inputs** |  |  |  |
| **NA Scale** | **0.62** | **\** | **\** |  | **NA Scale** | **0.56** | **\** | **\** |
| **NA Center** | **0.33** | **\** | **\** |  | **NA Center** | **0.36** | **\** | **\** |
| **Participant ID** | **0.29** | **\** | **\** |  | **Participant ID** | **0.34** | **\** | **\** |
| Encoder Length | 0.09 | \ | \ |  | Encoder Length | 0.06 | \ | \ |
| **12-hour interval** | **Importance** | **β** | **p** |  | **12-hour interval** | **Importance** | **β** | **p** |
| **Past Inputs** |  |  |  |  | **Past Inputs** |  |  |  |
| **Weekend or Weekday** | **0.14** | **-0.04** | **0.01** |  | **Weekend or Weekday** | **0.43** | **-0.04** | **0.01** |
| **Hours Since EMA1** | **0.13** | **0.02** | **0.22** |  | Max Screen-on Length | 0.07 | 0.00 | 0.95 |
| **NA (Own Past Value)** | **0.11** | **\** | **\** |  | N of Missed Outgoing Calls | 0.05 | -0.01 | 0.67 |
| N of Outgoing Calls | 0.09 | 0.01 | 0.38 |  | Percent Time in Rarely Visited Locations | 0.05 | -0.01 | 0.27 |
| Variance in Speed | 0.07 | 0.01 | 0.58 |  | Normalized Location Entropy | 0.04 | 0.00 | 0.72 |
| **Future Inputs** |  |  |  |  | **Future Inputs** |  |  |  |
| **N of Outgoing Calls** | **0.17** | **0.01** | **0.38** |  | **Hours Since EMA1** | **0.15** | **0.02** | **0.22** |
| **Weekend or Weekday** | **0.16** | **-0.04** | **0.01** |  | **Total Distance Traveled** | **0.12** | **0.01** | **0.40** |
| Percent Time of Valid GPS Data | 0.10 | 0.00 | 0.67 |  | **Weekend or Weekday** | **0.08** | **-0.04** | **0.01** |
| Max Length of Outgoing Calls | 0.09 | 0.00 | 0.92 |  | N of Incoming Calls | 0.07 | 0.01 | 0.24 |
| Hours Since EMA1 | 0.07 | 0.02 | 0.22 |  | Min Length of Outgoing Calls | 0.07 | -0.01 | 0.58 |
| **Static Inputs** |  |  |  |  | **Static Inputs** |  |  |  |
| **NA Scale** | **0.54** | **\** | **\** |  | **NA Scale** | **0.64** | **\** | **\** |
| **NA Center** | **0.36** | **\** | **\** |  | **Participant ID** | **0.33** | **\** | **\** |
| **Participant ID** | **0.36** | **\** | **\** |  | **NA Center** | **0.26** | **\** | **\** |
| Encoder Length | 0.07 | \ | \ |  | Encoder Length | 0.10 | \ | \ |
| **24-hour interval** | **Importance** | **β** | **p** |  | **24-hour interval** | **Importance** | **β** | **p** |
| **Past Inputs** |  |  |  |  | **Past Inputs** |  |  |  |
| **Weekend or Weekday** | **0.32** | **-0.04** | **0.01** |  | **Total Distance Traveled** | **0.13** | **0.02** | **0.20** |
| N of Location Transitions | 0.08 | -0.01 | 0.42 |  | **Weekend or Weekday** | **0.13** | **-0.04** | **0.01** |
| Average Speed | 0.06 | 0.03 | 0.12 |  | N of Location Transitions | 0.09 | -0.01 | 0.42 |
| Location Entropy | 0.05 | 0.00 | 0.78 |  | N of Missed Outgoing Calls | 0.07 | -0.01 | 0.61 |
| Min Length of Incoming Calls | 0.05 | 0.02 | 0.08 |  | N of Outgoing Calls | 0.06 | 0.03 | 0.14 |
| **Future Inputs** |  |  |  |  | **Future Inputs** |  |  |  |
| **Hours Since EMA1** | **0.14** | **0.02** | **0.22** |  | **Weekend or Weekday** | **0.58** | **-0.04** | **0.01** |
| Min Length of Incoming Calls | 0.10 | 0.02 | 0.08 |  | Total Screen-on Length | 0.08 | 0.03 | 0.09 |
| Total Distance Traveled | 0.09 | 0.02 | 0.20 |  | Total Distance Traveled | 0.05 | 0.02 | 0.20 |
| **Weekend or Weekday** | **0.09** | **-0.04** | **0.01** |  | Percent Time Stationary | 0.05 | -0.01 | 0.52 |
| Variance in Speed | 0.06 | 0.02 | 0.22 |  | Percent Time at Home (Self-report) | 0.04 | 0.00 | 0.80 |
| **Static Inputs** |  |  |  |  | **Static Inputs** |  |  |  |
| **NA Scale** | **0.63** | **\** | **\** |  | **NA Scale** | **0.69** | **\** | **\** |
| **Participant ID** | **0.34** | **\** | **\** |  | **NA Center** | **0.33** | **\** | **\** |
| **NA Center** | **0.27** | **\** | **\** |  | **Participant ID** | **0.24** | **\** | **\** |
| Encoder Length | 0.09 | \ | \ |  | Encoder Length | 0.07 | \ | \ |
| **48-hour interval** | **Importance** | **β** | **p** |  | **48-hour interval** | **Importance** | **β** | **p** |
| **Past Inputs** |  |  |  |  | **Past Inputs** |  |  |  |
| **Total Distance Traveled** | **0.17** | **0.01** | **0.76** |  | **Min Length of Outgoing Calls** | **0.24** | **0.00** | **0.93** |
| **Hours Since EMA1** | **0.12** | **0.02** | **0.22** |  | N of Correspondents Called | 0.08 | 0.00 | 0.89 |
| **Total Screen-on Length** | **0.10** | **-0.01** | **0.50** |  | Percent Time in Rarely Visited Locations | 0.07 | 0.01 | 0.65 |
| **Weekend or Weekday** | **0.07** | **-0.04** | **0.01** |  | N of GPS Data Points | 0.06 | -0.02 | 0.11 |
| N of Outgoing Calls | 0.06 | 0.00 | 0.97 |  | **Weekend or Weekday** | **0.06** | **-0.04** | **0.01** |
| **Future Inputs** |  |  |  |  | **Future Inputs** |  |  |  |
| **Weekend or Weekday** | **0.58** | **-0.04** | **0.01** |  | **Weekend or Weekday** | **0.24** | **-0.04** | **0.01** |
| Percent Time at Leisure Places (OSM) | 0.07 | 0.01 | 0.74 |  | **Mean Length of Outgoing Calls** | **0.10** | **0.00** | **0.97** |
| Total Distance Traveled | 0.05 | 0.01 | 0.76 |  | Percent Time at Work Places (Self-report) | 0.07 | 0.02 | 0.28 |
| N of Outgoing Calls | 0.05 | 0.00 | 0.97 |  | Variance in Speed | 0.06 | 0.02 | 0.44 |
| N of Missed Outgoing Calls | 0.04 | -0.02 | 0.40 |  | Min Length of Outgoing Calls | 0.05 | 0.00 | 0.93 |
| **Static Inputs** |  |  |  |  | **Static Inputs** |  |  |  |
| **NA Scale** | **0.64** | **\** | **\** |  | **NA Scale** | **0.56** | **\** | **\** |
| **NA Center** | **0.36** | **\** | **\** |  | **NA Center** | **0.37** | **\** | **\** |
| **Participant ID** | **0.26** | **\** | **\** |  | **Participant ID** | **0.32** | **\** | **\** |
| Encoder Length | 0.07 | \ | \ |  | Encoder Length | 0.08 | \ | \ |

*Note*. OSM = OpenStreetMap. Bold-type denotes importance over 0.10 or raw p < .05 in the bivariate linear mixed-effects model for that predictor. An asterisk (*) on the p-value denotes significance after Benjamini-Hochberg false discovery rate (FDR) correction across all bivariate tests for that outcome.

**Table S2.** Most important variables for temporal fusion transformers to predict momentary positive affect (PA).

| **Momentary PA Intensity**  **With Semantic Location Features**  **From OpenStreetMap** | | | |  | **Momentary PA Intensity**  **With Self-report Semantic Location Features** | | | |
| --- | --- | --- | --- | --- | --- | --- | --- | --- |
| **1-hour interval** | **Importance** | **β** | **p** |  | **1-hour interval** | **Importance** | **β** | **p** |
| **Past Inputs** |  |  |  |  | **Past Inputs** |  |  |  |
| **N of Outgoing Calls** | **0.18** | **-0.03** | **0.004** |  | **Weekend or Weekday** | **0.25** | **0.05** | **<0.001*** |
| **Weekend or Weekday** | **0.17** | **0.05** | **<0.001*** |  | **N of Significant Places** | **0.14** | **0.02** | **0.04** |
| **N of Missed Incoming Calls** | **0.16** | **-0.01** | **0.17** |  | Percent Time in Rarely Visited Locations | 0.07 | 0.01 | 0.53 |
| **N of Incoming Calls** | **0.08** | **-0.03** | **0.01** |  | Percent Time of Valid GPS Data | 0.04 | 0.00 | 0.92 |
| N of GPS Data Points | 0.07 | -0.01 | 0.47 |  | Percent Time at Leisure Places (Self-report) | 0.04 | 0.02 | 0.28 |
| **Future Inputs** |  |  |  |  | **Future Inputs** |  |  |  |
| **Weekend or Weekday** | **0.57** | **0.05** | **<0.001*** |  | **Weekend or Weekday** | **0.48** | **0.05** | **<0.001*** |
| **N of Missed Calls** | **0.12** | **-0.02** | **0.09** |  | **Percent Time of Valid GPS Data** | **0.15** | **0.00** | **0.92** |
| N of Missed Incoming Calls | 0.07 | -0.01 | 0.17 |  | N of Unlocked Episodes | 0.07 | 0.00 | 0.99 |
| Percent Time at Home (OSM) | 0.06 | -0.02 | 0.11 |  | N of Missed Incoming Calls | 0.06 | -0.01 | 0.17 |
| Percent Time at Leisure Places (OSM) | 0.05 | 0.02 | 0.21 |  | **N of Significant Places** | **0.05** | **0.02** | **0.04** |
| **Static Inputs** |  |  |  |  | **Static Inputs** |  |  |  |
| **PA Scale** | **0.70** | **\** | **\** |  | **PA Scale** | **0.60** | **\** | **\** |
| **Participant ID** | **0.31** | **\** | **\** |  | **Participant ID** | **0.35** | **\** | **\** |
| **PA Center** | **0.25** | **\** | **\** |  | **PA Center** | **0.28** | **\** | **\** |
| Encoder Length | 0.07 | \ | \ |  | Encoder Length | 0.10 | \ | \ |
| **3-hour interval** | **Importance** | **β** | **p** |  | **3-hour interval** | **Importance** | **β** | **p** |
| **Past Inputs** |  |  |  |  | **Past Inputs** |  |  |  |
| **Weekend or Weekday** | **0.57** | **0.05** | **<0.001*** |  | **Weekend or Weekday** | **0.40** | **0.05** | **<0.001*** |
| **PA (Own Past Value)** | **0.11** | **\** | **\** |  | **Radius of Gyration** | **0.07** | **0.02** | **0.04** |
| Hours Since EMA1 | 0.05 | -0.02 | 0.14 |  | **N of Correspondents Called** | **0.07** | **-0.03** | **0.009** |
| Percent Time at Home (OSM) | 0.04 | -0.02 | 0.21 |  | N of Location Transitions | 0.06 | 0.01 | 0.67 |
| Max Length of Incoming Calls | 0.04 | -0.01 | 0.22 |  | **Normalized Location Entropy** | **0.05** | **0.03** | **0.02** |
| **Future Inputs** |  |  |  |  | **Future Inputs** |  |  |  |
| **Weekend or Weekday** | **0.18** | **0.05** | **<0.001*** |  | **Percent Time at Leisure Places (Self-report)** | **0.17** | **0.02** | **0.20** |
| **Normalized Location Entropy** | **0.13** | **0.03** | **0.02** |  | **N of Location Transitions** | **0.17** | **0.01** | **0.67** |
| Min Screen-on Length | 0.09 | 0.00 | 0.81 |  | **Weekend or Weekday** | **0.11** | **0.05** | **<0.001*** |
| Max Length of Incoming Calls | 0.08 | -0.01 | 0.22 |  | **Normalized Location Entropy** | **0.06** | **0.03** | **0.02** |
| N of GPS Data Points | 0.07 | -0.01 | 0.60 |  | N of Incoming Calls | 0.06 | -0.02 | 0.08 |
| **Static Inputs** |  |  |  |  | **Static Inputs** |  |  |  |
| **PA Scale** | **0.63** | **\** | **\** |  | **PA Scale** | **0.66** | **\** | **\** |
| **Participant ID** | **0.33** | **\** | **\** |  | **PA Center** | **0.30** | **\** | **\** |
| **PA Center** | **0.27** | **\** | **\** |  | **Participant ID** | **0.29** | **\** | **\** |
| Encoder Length | 0.09 | \ | \ |  | Encoder Length | 0.09 | \ | \ |
| **6-hour interval** | **Importance** | **β** | **p** |  | **6-hour interval** | **Importance** | **β** | **p** |
| **Past Inputs** |  |  |  |  | **Past Inputs** |  |  |  |
| **Weekend or Weekday** | **0.24** | **0.05** | **<0.001*** |  | **Max Screen-on Length** | **0.36** | **0.00** | **0.66** |
| **N of Missed Calls** | **0.13** | **0.00** | **0.85** |  | **Total Distance Traveled** | **0.12** | **0.03** | **0.03** |
| **Max Screen-on Length** | **0.11** | **0.00** | **0.66** |  | **Weekend or Weekday** | **0.11** | **0.05** | **<0.001*** |
| **Location Entropy** | **0.08** | **0.06** | **<0.001*** |  | Percent Time at Leisure Places (Self-report) | 0.05 | 0.03 | 0.16 |
| Percent Time Stationary | 0.06 | -0.02 | 0.18 |  | Percent Time at Home (Self-report) | 0.05 | -0.02 | 0.22 |
| **Future Inputs** |  |  |  |  | **Future Inputs** |  |  |  |
| **Min Screen-on Length** | **0.15** | **0.00** | **0.96** |  | **Variance in Speed** | **0.26** | **0.01** | **0.44** |
| **N of Significant Places** | **0.15** | **0.05** | **<0.001*** |  | **Weekend or Weekday** | **0.13** | **0.05** | **<0.001*** |
| N of Correspondents Called | 0.09 | -0.02 | 0.06 |  | **Max Length of Outgoing Calls** | **0.13** | **-0.01** | **0.55** |
| **Weekend or Weekday** | **0.09** | **0.05** | **<0.001*** |  | **Max Length of Incoming Calls** | **0.10** | **-0.01** | **0.31** |
| **N of Unlocked Episodes** | **0.06** | **0.03** | **0.04** |  | **Location Entropy** | **0.10** | **0.06** | **<0.001*** |
| **Static Inputs** |  |  |  |  | **Static Inputs** |  |  |  |
| **PA Scale** | **0.65** | **\** | **\** |  | **PA Scale** | **0.65** | **\** | **\** |
| **PA Center** | **0.34** | **\** | **\** |  | **PA Center** | **0.31** | **\** | **\** |
| **Participant ID** | **0.24** | **\** | **\** |  | **Participant ID** | **0.30** | **\** | **\** |
| Encoder Length | 0.10 | \ | \ |  | Encoder Length | 0.07 | \ | \ |
| **12-hour interval** | **Importance** | **β** | **p** |  | **12-hour interval** | **Importance** | **β** | **p** |
| **Past Inputs** |  |  |  |  | **Past Inputs** |  |  |  |
| **Hours Since EMA1** | **0.24** | **-0.02** | **0.14** |  | **Weekend or Weekday** | **0.51** | **0.05** | **<0.001*** |
| **N of GPS Data Points** | **0.11** | **0.00** | **0.84** |  | **Percent Time in Rarely Visited Locations** | **0.09** | **0.04** | **0.008** |
| **Weekend or Weekday** | **0.10** | **0.05** | **<0.001*** |  | N of Incoming Calls | 0.08 | 0.00 | 0.71 |
| **PA (Own Past Value)** | **0.10** | **\** | **\** |  | Max Screen-on Length | 0.04 | -0.02 | 0.09 |
| N of Outgoing Calls | 0.09 | -0.01 | 0.64 |  | N of GPS Data Points | 0.04 | 0.00 | 0.84 |
| **Future Inputs** |  |  |  |  | **Future Inputs** |  |  |  |
| **N of Outgoing Calls** | **0.24** | **-0.01** | **0.64** |  | **N of Incoming Calls** | **0.13** | **0.00** | **0.71** |
| **Weekend or Weekday** | **0.18** | **0.05** | **<0.001*** |  | **Hours Since EMA1** | **0.11** | **-0.02** | **0.14** |
| Max Length of Incoming Calls | 0.07 | 0.00 | 0.87 |  | **Total Distance Traveled** | **0.10** | **0.03** | **0.04** |
| N of Correspondents Called | 0.06 | -0.01 | 0.64 |  | **Weekend or Weekday** | **0.07** | **0.05** | **<0.001*** |
| Percent Time of Valid GPS Data | 0.05 | 0.00 | 0.96 |  | Min Length of Outgoing Calls | 0.06 | 0.01 | 0.38 |
| **Static Inputs** |  |  |  |  | **Static Inputs** |  |  |  |
| **PA Scale** | **0.58** | **\** | **\** |  | **PA Scale** | **0.69** | **\** | **\** |
| **Participant ID** | **0.34** | **\** | **\** |  | **Participant ID** | **0.30** | **\** | **\** |
| **PA Center** | **0.34** | **\** | **\** |  | **PA Center** | **0.24** | **\** | **\** |
| Encoder Length | 0.07 | \ | \ |  | **Encoder Length** | **0.10** | **\** | **\** |
| **24-hour interval** | **Importance** | **β** | **p** |  | **24-hour interval** | **Importance** | **β** | **p** |
| **Past Inputs** |  |  |  |  | **Past Inputs** |  |  |  |
| **Weekend or Weekday** | **0.47** | **0.05** | **<0.001*** |  | **Hours Since EMA1** | **0.14** | **-0.02** | **0.14** |
| N of Location Transitions | 0.10 | 0.02 | 0.11 |  | **Weekend or Weekday** | **0.12** | **0.05** | **<0.001*** |
| N of Missed Outgoing Calls | 0.07 | -0.01 | 0.56 |  | Total Distance Traveled | 0.10 | 0.01 | 0.59 |
| **Min Length of Incoming Calls** | **0.05** | **-0.03** | **0.006** |  | **Percent Time at Work Places (Self-report)** | **0.09** | **-0.04** | **0.005** |
| Total Distance Traveled | 0.05 | 0.01 | 0.59 |  | N of Unlocked Episodes | 0.06 | -0.01 | 0.49 |
| **Future Inputs** |  |  |  |  | **Future Inputs** |  |  |  |
| **Min Length of Incoming Calls** | **0.11** | **-0.03** | **0.006** |  | **Weekend or Weekday** | **0.57** | **0.05** | **<0.001*** |
| **Hours Since EMA1** | **0.10** | **-0.02** | **0.14** |  | Total Distance Traveled | 0.07 | 0.01 | 0.59 |
| **Weekend or Weekday** | **0.10** | **0.05** | **<0.001*** |  | Percent Time at Home (Self-report) | 0.05 | -0.01 | 0.35 |
| Total Distance Traveled | 0.09 | 0.01 | 0.59 |  | Percent Time Stationary | 0.05 | -0.02 | 0.12 |
| Max Length of Incoming Calls | 0.07 | -0.03 | 0.06 |  | **Total Screen-on Length** | **0.05** | **-0.04** | **0.007** |
| **Static Inputs** |  |  |  |  | **Static Inputs** |  |  |  |
| **PA Scale** | **0.70** | **\** | **\** |  | **PA Scale** | **0.67** | **\** | **\** |
| **Participant ID** | **0.29** | **\** | **\** |  | **PA Center** | **0.34** | **\** | **\** |
| **PA Center** | **0.23** | **\** | **\** |  | **Participant ID** | **0.25** | **\** | **\** |
| **Encoder Length** | **0.11** | **\** | **\** |  | Encoder Length | 0.07 | \ | \ |
| **48-hour interval** | **Importance** | **β** | **p** |  | **48-hour interval** | **Importance** | **β** | **p** |
| **Past Inputs** |  |  |  |  | **Past Inputs** |  |  |  |
| **Hours Since EMA1** | **0.14** | **-0.02** | **0.14** |  | **Min Length of Outgoing Calls** | **0.27** | **-0.02** | **0.17** |
| **Total Distance Traveled** | **0.11** | **-0.01** | **0.65** |  | Percent Time in Rarely Visited Locations | 0.09 | 0.02 | 0.31 |
| N of Outgoing Calls | 0.09 | -0.03 | 0.05 |  | Percent Time at Home (Self-report) | 0.09 | -0.02 | 0.29 |
| **Weekend or Weekday** | **0.08** | **0.05** | **<0.001*** |  | **Weekend or Weekday** | **0.07** | **0.05** | **<0.001*** |
| Total Screen-on Length | 0.08 | 0.00 | 0.91 |  | **N of Incoming Calls** | **0.06** | **-0.04** | **0.03** |
| **Future Inputs** |  |  |  |  | **Future Inputs** |  |  |  |
| **Weekend or Weekday** | **0.64** | **0.05** | **<0.001*** |  | **Weekend or Weekday** | **0.24** | **0.05** | **<0.001*** |
| Percent Time at Leisure Places (OSM) | 0.07 | 0.02 | 0.50 |  | **Percent Time at Work Places (Self-report)** | **0.07** | **-0.03** | **0.03** |
| N of Missed Outgoing Calls | 0.05 | -0.03 | 0.09 |  | Mean Length of Outgoing Calls | 0.07 | -0.02 | 0.24 |
| Total Distance Traveled | 0.05 | -0.01 | 0.65 |  | Total Screen-on Length | 0.07 | 0.00 | 0.91 |
| Percent Time Stationary | 0.03 | -0.01 | 0.44 |  | N of Unlocked Episodes | 0.06 | -0.01 | 0.50 |
| **Static Inputs** |  |  |  |  | **Static Inputs** |  |  |  |
| **PA Scale** | **0.69** | **\** | **\** |  | **PA Scale** | **0.62** | **\** | **\** |
| **PA Center** | **0.34** | **\** | **\** |  | **Participant ID** | **0.33** | **\** | **\** |
| **Participant ID** | **0.24** | **\** | **\** |  | **PA Center** | **0.30** | **\** | **\** |
| Encoder Length | 0.07 | \ | \ |  | Encoder Length | 0.07 | \ | \ |

*Note*. OSM = OpenStreetMap. Bold-type denotes importance over 0.10 or raw p < .05 in the bivariate linear mixed-effects model for that predictor. An asterisk (*) on the p-value denotes significance after Benjamini-Hochberg false discovery rate (FDR) correction across all bivariate tests for that outcome.

**Table S3.** TFT’s performance in predicting discrete affect on the test dataset (*n*=133 individuals; *N*=532 surveys).

| Discrete Affect | Model | Feature sets | R² (%) [95% CI] | MAE [95% CI] |
| --- | --- | --- | --- | --- |
| Frustrated | TFT | 6h+OSM+Self-report locations | 16.7 [5.5,27.8] | 0.97 [0.85,1.10] |
| Sad | TFT | 6h+OSM+Self-report locations | 23.2 [6.0,37.1] | 0.67 [0.55,0.79] |
| Sluggish | TFT | 6h+OSM+Self-report locations | 27.0 [13.5, 38.3] | 1.03 [0.91, 1.14] |
| Worried | TFT | 6h+OSM+Self-report locations | 26.1 [13.0, 37.5] | 0.87 [0.77,0.98] |
| Content | TFT | 1h+OSM+Self-report locations | 27.0 [9.9, 39.5] | 0.91 [0.83,1.00] |
| Enthusiastic | TFT | 1h+OSM+Self-report locations | 0.19 [-3.2, 35.8] | 1.03 [0.92,1.16] |
| Happy | TFT | 1h+OSM+Self-report locations | 27.5 [0.08, 42.0] | 0.96 [0.88, 1.07] |

*Note.* MAE = Mean Absolute Error; OSM = OpenStreetMap; TFT = Temporal Fusion Transformer. R² and MAE are computed on the held-out test set; brackets denote the 95% participant-level bootstrap confidence interval (2,000 resamples with replacement).
